# Interspecies chimeric conditions affect the developmental rate of human pluripotent stem cells

**DOI:** 10.1371/journal.pcbi.1008778

**Published:** 2021-03-01

**Authors:** Jared Brown, Christopher Barry, Matthew T. Schmitz, Cara Argus, Jennifer M. Bolin, Michael P. Schwartz, Amy Van Aartsen, John Steill, Scott Swanson, Ron Stewart, James A. Thomson, Christina Kendziorski

**Affiliations:** 1 Department of Statistics, University of Wisconsin-Madison, Wisconsin, United States of America; 2 Morgridge Institute for Research, Madison, Wisconsin, United States of America; 3 NSF Center for Sustainable Nanotechnology, Department of Chemistry, University of Wisconsin-Madison, Wisconsin, United States of America; 4 Department of Cell and Regenerative Biology, University of Wisconsin School of Medicine and Public Health, Madison, Wisconsin, United States of America; 5 Department of Molecular, Cellular, and Developmental Biology, University of California, Santa Barbara, California, United States of America; 6 Department of Biostatistics and Medical Informatics, University of Wisconsin-Madison, Wisconsin, United States of America; EMBL-Heidelberg, GERMANY

## Abstract

Human pluripotent stem cells hold significant promise for regenerative medicine. However, long differentiation protocols and immature characteristics of stem cell-derived cell types remain challenges to the development of many therapeutic applications. In contrast to the slow differentiation of human stem cells *in vitro* that mirrors a nine-month gestation period, mouse stem cells develop according to a much faster three-week gestation timeline. Here, we tested if co-differentiation with mouse pluripotent stem cells could accelerate the differentiation speed of human embryonic stem cells. Following a six-week RNA-sequencing time course of neural differentiation, we identified 929 human genes that were upregulated earlier and 535 genes that exhibited earlier peaked expression profiles in chimeric cell cultures than in human cell cultures alone. Genes with accelerated upregulation were significantly enriched in Gene Ontology terms associated with neurogenesis, neuron differentiation and maturation, and synapse signaling. Moreover, chimeric mixed samples correlated with *in utero* human embryonic samples earlier than human cells alone, and acceleration was dose-dependent on human-mouse co-culture ratios. The altered gene expression patterns and developmental rates described in this report have implications for accelerating human stem cell differentiation and the use of interspecies chimeric embryos in developing human organs for transplantation.

## Author summary

Human pluripotent stem cells often require long *in vitro* protocols to form mature cell types of clinical relevance for potential regenerative therapies, a ramification of a nine-month developmental clock *in utero* that also runs *ex utero*. What controls species-specific developmental time and whether the timer is amenable to acceleration is unknown. Further, interspecies chimeric embryos are increasingly being created to study early human development or explore the potential growth of human organs for transplantation. How the conflicting developmental speeds of cells from different species co-differentiating together affect each other is not understood. Here, using genome-wide transcriptional analysis of RNA-sequencing time courses, we show that 1) co-differentiating human embryonic stem cells intermixed with mouse stem cells accelerated elements of human developmental programs, 2) the acceleration was dose-dependent on the proportion of mouse cells, and 3) human cells in chimeric samples correlated to *in utero* samples earlier than human only samples. Our results provide evidence that some components of species-specific developmental clocks may be susceptible to acceleration.

## Introduction

Mammals develop at tremendously different rates *in utero*, however little is known about the mechanisms regulating species-specific developmental speeds. Curiously, when pluripotent stem cells are cultured *in vitro*, they retain the developmental timing of their species of origin despite the lack of maternal factors, suggesting the existence of an intrinsic developmental clock [1–7]. Currently, the nature of the species-specific developmental clock, including the extent to which it can be altered, is unknown [8]. The retention of a slow differentiation rate that reflects a nine-month human gestation timeline often results in long differentiation protocols and immature cell characteristics that impede many potential clinical applications of human pluripotent stem cells [9,10].

In contrast to the slow differentiation of human stem cells, mouse stem cells differentiate substantially more quickly, reflecting a 20-day rather than a nine-month gestation timeline [5,11,12]. For example, mature neurons are produced in only 5–14 days from mouse ES cells, while the same cell types can take several months to generate from human embryonic stem (hES) cells [7,13–15]. It remains unknown if the developmental pace of one species can influence that of another. Previously, we found that hES cell differentiation was not accelerated in teratomas developed in a mouse despite being exposed to murine host factors [1]. However, we did not test whether factors active during murine embryonic development could be sufficient to accelerate hES cell differentiation.

Here, we investigated whether hES cells co-differentiated among mouse pluripotent stem cells could accelerate their developmental rate. Under neural differentiation of chimeric co-cultures, we found earlier upregulation and peak expression of hundreds of genes involved in neurogenesis, neuron maturation, and synapse signaling compared to hES cells alone. The accelerated effects were dose-dependent on the starting ratios of human-mouse cells in co-cultures, and chimeric cultures correlated to *in utero* human embryonic samples earlier than human cells alone. We also describe temporal differences in gene expression levels related to cell type and brain region identity, suggesting there may be other nuanced effects on gene expression from chimeric co-culture conditions. Overall, we demonstrate that chimeric human-mouse culture conditions are sufficient to accelerate elements of human stem cell differentiation.

## Results

### Comprehensive RNA-sequencing time course of neural differentiation in chimeric human-mouse co-cultures

**Fig 1:**
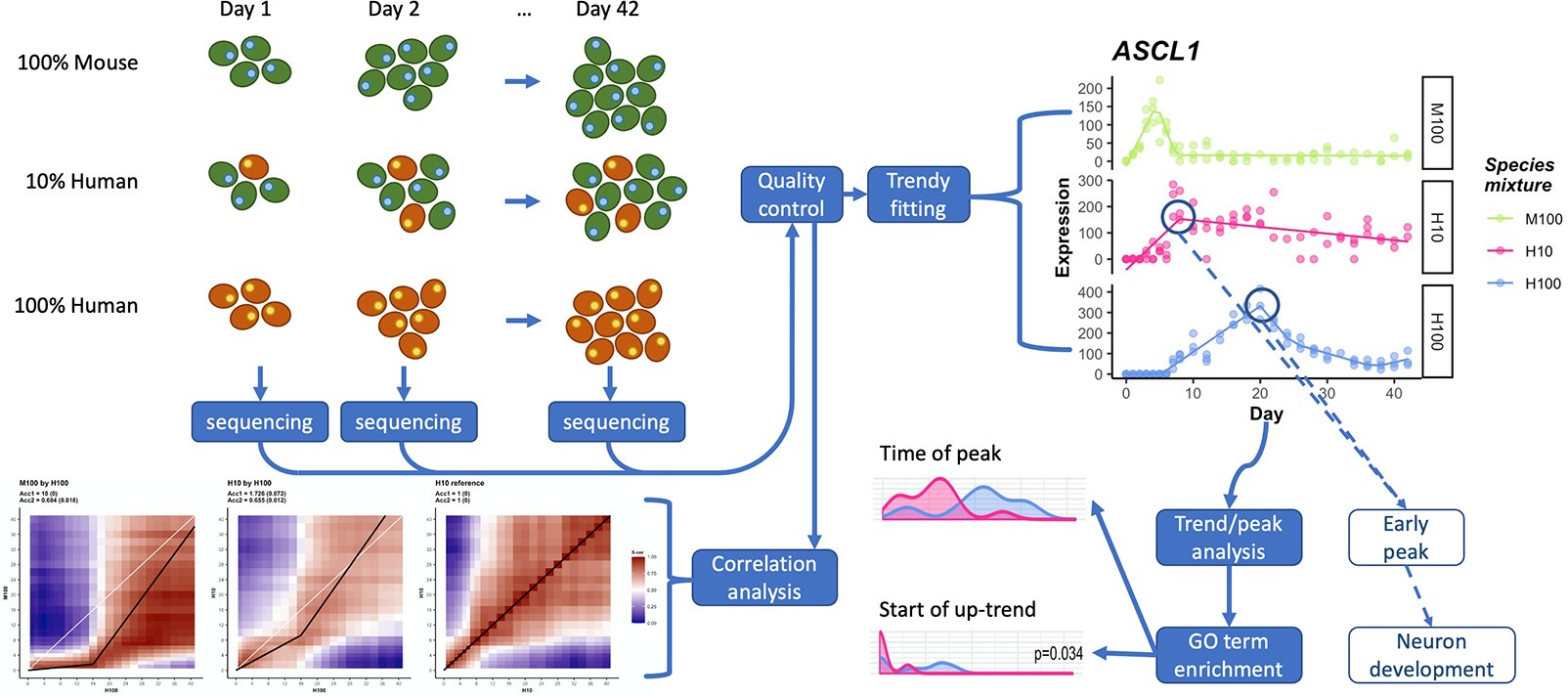
Overview of data collection/analysis pipeline. (top left) Human (red) and mouse (green) cells are cultured at various mixing proportions over a 42 day neural differentiation time course, harvesting samples every 1–2 days for RNA-seq. Low quality biological replicates are removed from analysis and the data are normalized. (top right) Normalized data are fit to segmented regression built for RNA-seq data (Trendy) and temporal gene characteristics, such as peak times, are identified. (bottom right) Classified gene sets are further analyzed, including enrichment analysis for GO terms which are temporally accelerated or otherwise systematically altered in H10 compared to H100. (bottom left) In parallel to the previous analysis, normalized data are also correlated between time courses to identify transcriptome-wide effects.

We previously described a detailed RNA-sequencing (RNA-seq) time course of mouse and human pluripotent stem cells over three- or six-weeks of neural differentiation, respectively, to characterize the drastically different species-specific rates of development *in vitro* [1]. Here, we set out to determine if co-differentiating human cells with mouse cells together could induce the human cells to differentiation at a quickened pace. Since hES cells are thought to more closely represent a post-implantation pluripotent stage, we used the similarly-staged mouse Epiblast stem (mEpiS) cells to compare with H9 hES cells [16–18]. To identify cells from each species, we used mEpiS cells constitutively expressing cytoplasmic efficient green fluorescent protein (EGFP) and H9 cells expressing nuclear-localized H2B-mCherry (Fig 1).

To maximize any potential mouse-induced effects on human differentiation rate, we began by outnumbering human cells with the more quickly differentiating mouse cells in a ten-to-one ratio. 10% human co-cultured cells (H10), along with 100% mouse (M100) or 100% human (H100) control samples, were cultured under identical neural differentiation culture conditions (see Materials and Methods) and samples in triplicate were collected for RNA-seq every 24 or 48 hours for six weeks (Fig 1). To minimize any confounding of results with known differences in cell cycle and cell fate choices due to differences in cell densities [19–23], interspecies cell seeding confluencies were kept constant across species mixtures. After aligning transcripts to a combined human-mouse transcriptome to derive species-specific expression from the chimeric samples, samples passing quality control parameters (S1 Fig, see Materials and Methods) were processed for correlation analysis, fitted with gene expression patterns using the segmentation regression analysis R-package Trendy [24], and the timing of expression pattern changes were compared across samples (Fig 1).

**Fig 2:**
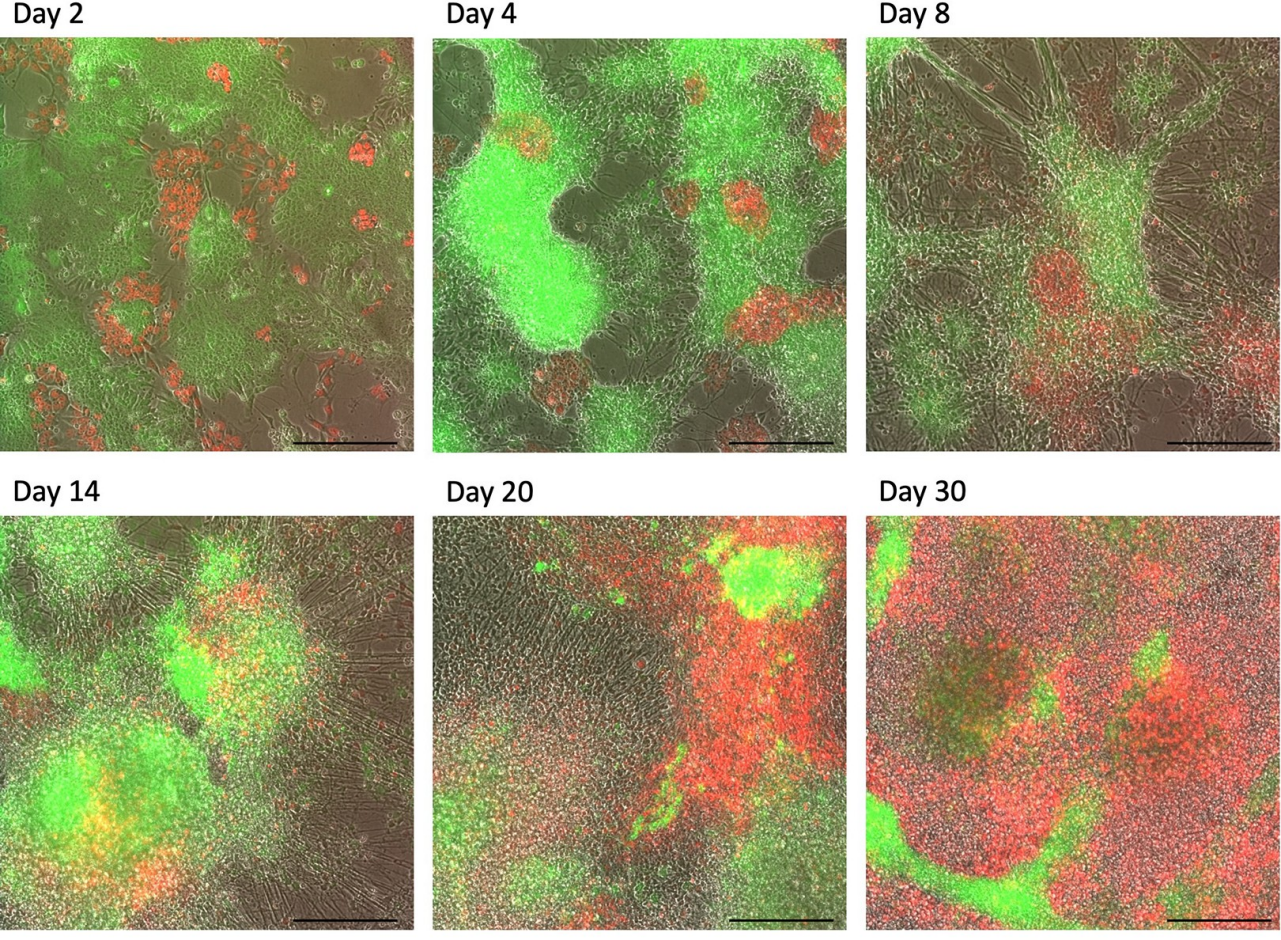
Microscopy images of the H10 mixture across the time course. 10% Human ES cells expressing nuclear-localized H2B-mCherry (red) were mixed with 90% mouse EpiS cells (expressing cytoplasmic GFP (green)) and co-cultured together under neural differentiation conditions for six weeks. Images captured at the time points indicated show clusters of associating human and mouse cells (red and green clusters respectively). Scale bars = 200μm.

Although mouse and human cells were singularized before seeding, time lapse microscopy revealed that, despite the clear occurrence of interspecies cell-cell interactions, cells preferentially clustered and proliferated with cells of their own species (Fig 2 and S1 and S2 Movies). Flow cytometry analysis revealed that while the intended starting cell ratios were seeded, as mouse cells differentiated quickly to become post-mitotic neurons, the still-proliferating human progenitor cells eventually overtook the culture. By day 12 of differentiation ~50% of H10 samples were of human composition, and by day 16 over 75% of samples were human cells (S2 Fig).

### Human neurogenic and synaptic genes were upregulated earlier in human-mouse chimeric co-cultures

**Fig 3:**
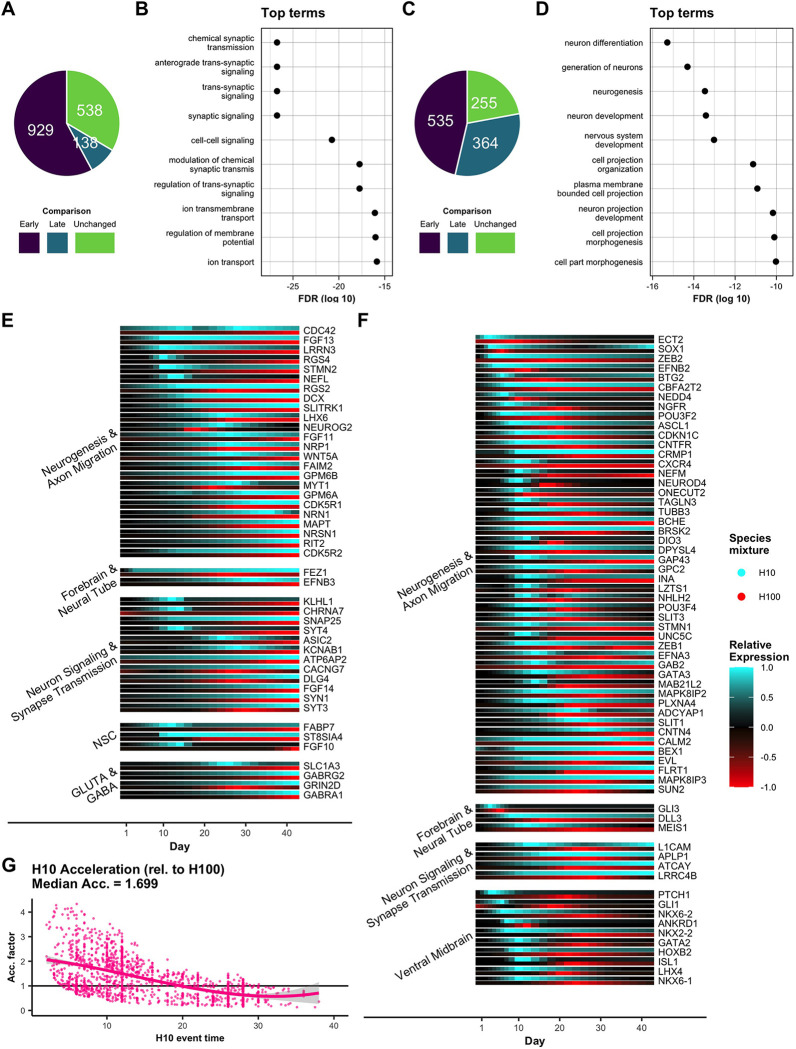
Changes in neurodevelopmental gene expression are accelerated in human ES cells differentiated among mouse EpiS cells. (A) All genes which trend up in both H10 and H100 are classified as either early, late, or unchanged in H10 relative to H100 (omitting genes which already start up-trending at day 0 in both H10 and H100). (B) The top 10 most significant GO terms enriched for early upregulation in H10 demonstrated a clear pattern of acceleration in neuron and synaptic signaling-related genes (term enrichments shown as log10 adjusted p-values (FDR)). (C) All genes which peak in both H10 and H100 were classified as either early, late, or unchanged in H10 relative to H100. (D) The top 10 most significant GO terms enriched for early peaks in H10 showed acceleration of genes involved in neurogenesis and neuron development (term enrichments shown as log10 adjusted p-values (FDR)). (E) Relative expression plots of a curated subset of early-up (EU) genes collected into functional/regional groups. H10 (blue) and H100 (red) time courses were scaled such that 0 expression shows black and maximum expression between H10 and H100 shows as 1/-1 (within gene). (F) Relative expression plots of a curated subset of early-peak (EP) genes collected into functional/regional groups. (G) Genes with shared peaks or shared up-trends between H10 and H100 were used to compute acceleration rate point estimates as the ratio of H100 event times (e.g., time of peak in H100) to H10 event times. Point estimates were smoothed to give a continuous estimate of the H10 acceleration factor. The median fitted acceleration factor calculated over the first 16 days for H10 was given as 1.699.

To determine if gene expression patterns were accelerated in chimeric co-cultures, genes with fitted expression trends were compared between neural differentiation of human cells alone (H100) versus cells in a co-culture of 10% human cells mixed with 90% mouse cells (H10). We first asked if upregulated genes (genes trending up immediately or genes showing no change and then trending up) were upregulated earlier in mixed compared to control samples. Our bioinformatic analysis revealed that 929 genes were upregulated significantly earlier (S1 Data) (begin up trending at least 2 days earlier) in H10 versus H100 samples, representing over 57% of all genes that trend up in both H10 and H100, excluding genes that begin to trend up on day 0 in both cases (Fig 3A). We recognized several well-described neurogenic genes identified as accelerated in this early-upregulated category (S3A Fig), including genes involved in neural differentiation and migration (e.g., *STMN2*, *DCX*, *NEFL*, *NEUROG2*, *MYT1*, *MAPT*), neuronal signaling and synapse transmission (e.g., *SNAP25*, *SYT3*, *SYT4*, *SYN1*), neural stem cell identity (e.g., *FABP7*, *FGF10*), and glutamatergic and GABAergic neurons (e.g., *SLC1A3*, *GRIN2D*, *GABRA1*; Fig 3E). Therefore, genes from a seemingly wide range of neurodevelopmental functions were upregulated earlier under chimeric differentiation conditions.

Given that several recognizable neurogenic genes were among those identified as upregulated earlier in H10 compared to H100 samples (Figs 3E and S3A), we set out to statistically test if early upregulated genes were specific to neural differentiation or a collection of genes within a random assortment of cellular processes. Functional GO-term enrichment of early-upregulated genes revealed that all of the ten most statistically significantly-enriched terms were associated with neuron and synaptic signaling (Fig 3B). In contrast, we did not observe neural-related GO term enrichment in genes upregulated later in H10 than in H100 (S4A Fig), confirming that neural genes were indeed specifically upregulated earlier in human cells co-differentiated with mouse cells.

In addition to the earlier upregulation of genes associated with neuron and synapse signaling, the duration of up-regulation was also significantly longer, often still trending upwards at the end point of the 6-week time course (S5A Fig). However, despite earlier onset of upregulation, their slopes were also significantly less steep than those of H100 samples (S5A Fig). These results indicate an earlier onset of synaptic signaling gene activation characterized by a more sustained, yet slower, rate of upregulation.

To ensure these results were not artifacts of transcript misalignment to the wrong species within the combined human and mouse reference transcriptome, we conducted an additional interspecies mixing time course experiment where intermixed cells were re-purified according to their species using Fluorescence-Activated Cell Sorting (FACS) (human-mCherry vs mouse-GFP) at each time point prior to RNA-isolation and sequencing. Importantly, post-sorted samples were aligned to the identical combined human and mouse transcriptome library. Sorted (s) sample datasets are therefore labeled sH100, sH10, and sM100 to differentiate the sorted samples from the previously described data.

Computation of empirical misalignment rates showed low overall rates of misalignment across days (median 0.53% for sH100 and median 2.23% for sH10) (S6A Fig), and enrichment of misaligned transcripts failed to demonstrate any bias in neural-associated genes (S6B Fig). Although the sorted time course could not be performed in triplicate, nor at the same sampling frequency as the unsorted time course due to the extensive sort times necessary to collect enough cells to achieve sufficient read-depth, sorted sample expression analyses resulted in the same acceleration effects in sH10 relative to sH100 that we observed in unsorted samples (S6C–S6E Fig), confirming that our earlier detection of acceleration was not due to species-misaligned transcripts.

### Regulation of peak gene expression profiles occurs more rapidly in co-cultures with mouse stem cells

During development, genes involved in neural differentiation are often not simply turned on, but rather are expressed in temporally-regulated dynamic patterns [25,26]. To determine if genes with coordinated expression profiles were regulated more quickly, we next tested whether genes with peak expression profiles (consecutive up-down or up-flat segments) peaked earlier under chimeric versus human control conditions.

Overall, we identified 535 genes that peaked earlier (at least two days) (S1 Data) in chimeric culture conditions compared to control samples, representing over 46% of all peaking genes identified in the time course (Fig 3C). Similarly to early-upregulated genes, we recognized several peaking genes involved in neural development in the accelerated peak category (Figs 3F and S3B), including genes involved in neurogenesis (e.g., *ASCL1*, *NGFR*, *NEFM*, *TUBB3*), neural tube development (e.g., *MEIS1*, *GLI3*, *DLL3*), neuron signaling (e.g., *SNAP25*, *ATCAY*), and ventral midbrain differentiation (e.g., *ISL1*, *LHX4*, *NKX6-1*). We further validated that genes involved in neurodevelopment were specifically peaking early through GO-term enrichment analysis, and we found that all of the top ten most significantly enriched terms were associated with neural development (Fig 3D), whereas no obvious trend in neural-related GO-terms was found for genes with delayed peaks (S4B Fig). In contrast to early-upregulated genes that were enriched in neuron and synaptic signaling, early peaked genes were involved in neurogenesis, neuron projection development, and neuron differentiation (Fig 3D). Further, whereas early-upregulated genes had a slower rate of increase compared to control cells, early peaked genes exhibited an earlier time of start of upregulation towards the peak and a faster rate of upregulation to reach the peak (S5B Fig). Taken together, we report that the regulation of neurogenic genes was specifically accelerated in H10 compared to H100.

To quantify the degree of acceleration and investigate if acceleration was variable or uniform across the time course, we considered genes with shared peaks or shared up trends in both H10 and H100 and computed acceleration factors as the percent difference in time to peak or the start of up regulation in H10 compared to H100. Smooth regression of these point estimates provided a continuous estimate of the relative acceleration between H10 and H100 (Fig 3G, see Materials and Methods).

From this analysis, we uncovered that the majority of acceleration was in fact not constant over the course of co-differentiation. Rather, the majority of acceleration takes place during the first 16 days. While the median acceleration factor (reflecting fold-change acceleration of expression events) during this time was 1.699, acceleration varied from a maximum factor of 2.75 at the earliest stages of differentiation, converging eventually to a factor of 1 (non-accelerated) by day 20 (Fig 3G). It is notable that this gradual reduction in acceleration rate occurs concurrently as human cells begin out-proliferating post-mitotic mouse neurons (Figs 2 and S2). Human cells start outnumbering mouse cells at day 12, the time at which acceleration effects dissipate, suggesting a correlation between mouse cell number and the acceleration effect they induce in co-culture (Figs 3G and S2).

### Mouse gene expression patterns were decelerated under chimeric conditions

We next wondered if developmental time warping was a bi-direction effect on both species during chimeric conditions (i.e., that mouse cell development was slowed while that of human cells was accelerated). We therefore conducted a similar interspecies time course, this time outnumbering mouse cells with human cells (85% hES cells vs 15% mEpiS cells; H85 = M15) (S2 Data).

Indeed, the same analysis pipeline that identified accelerated patterns of human gene expression in chimeric conditions resulted in an opposite, deceleration, effect observed for mouse genes. We observed 87 genes which were upregulated later in M15 than in M100 (23.6% of genes with shared up trends, excluding genes which start trending up on day 0 in both species mixtures) and 562 genes which peak later in M15 than in M100 (58.2% of genes with shared peaks) (S7A and S7B Fig). Further, when acceleration was measured by comparing genes with shared up trends and peaks, a median acceleration factor of 0.894 was measured over the first 16 days, indicating a deceleration of M15 relative to M100 (S7C Fig). Enrichment of the subset of genes which were upregulated or peaked later in M15 shows neural terms to be either uniquely enriched for late upregulation or more significantly enriched for late peaks (S7D–S7G Fig). Together with the results among human aligned expression, this indicates that the interspecies co-culture influence was bi-directional, affecting both human and mouse cells. This may also suggest that common mechanisms may regulate developmental time across species.

### Chimeric co-culture affected the timing and expressions levels of some genes associated with neuron or brain region identity

Our neural differentiation protocol recapitulates a general neural developmental program and produces neurons of various regional identities [1]. To determine if chimeric co-culture of hES cells would affect cell lineage outcomes, we identified genes that were most differentially expressed (S1 Data) (measured as fold change between maximum expression along the time course) in chimeric mixed samples compared to hES cell controls (S8 Fig).

We observed some changes in the expression of transient signals as well as changes in sustained region-specific expression. For example, certain genes associated with the anterior dorsal neural tube showed earlier downregulation in H10 compared to H100, whereas genes linked to Gluta- and GABAergic neurons and neuron signal transduction showed patterns of downregulation at later times (S8 Fig). Other genes broadly associated with neurogenesis show a mixture of these patterns. In contrast, some genes associated with the ventral midbrain showed transient upregulation in chimeric mixed samples compared to control samples (S8 Fig). These effects would be consistent with an early exposure of *Shh* from mouse cells that could have triggered a cascade of downstream effects on gene expression, including *FOXA2* [27], *NKX2*.*1*, and *PHOX2B* (S8 Fig) [28,29]. Our analysis therefore revealed that some genes associated with neuron cell type and regional identity were temporally and/or differentially expressed under chimeric conditions.

To verify that the acceleration effects described in this report were not largely due to a general shift towards neural cell types that appear earlier in development rather than a true acceleration, we performed a deconvolution analysis of H100 and H10 samples to monitor the appearance of various progenitor and intermediate cell stages and their differentiation over time. This type of analysis estimates the relative proportions of cell types that may be present in a bulk sample by comparing bulk expression to a reference of purified or annotated single cell data. We compared our data to the CoDEx dataset of annotated single-cell sequencing from the developing human cortex [30] with the MuSiC R package [31]. Smoothed estimates of neural stage proportions (see Materials and Methods) indicated that co-cultured human cells mirrored the developmental progression of cell types of control samples, but at an accelerated pace (S9 Fig). Specifically, similar progenitor-to-mature neural cell markers appeared in the same order in H10 and H100 (S9 Fig), yet high proportions of excitatory neurons in H10 occurred earlier (days 12–16) compared to H100 (days 18–24). Taken together, although differential expression analyses identified changes in expression levels of some genes implicated in nervous system development, differentiation followed similar lineage pathways but at accelerated rates in chimeric compared to unmixed conditions.

### Acceleration effects are dose-dependent on percentage of mouse stem cells

**Fig 4:**
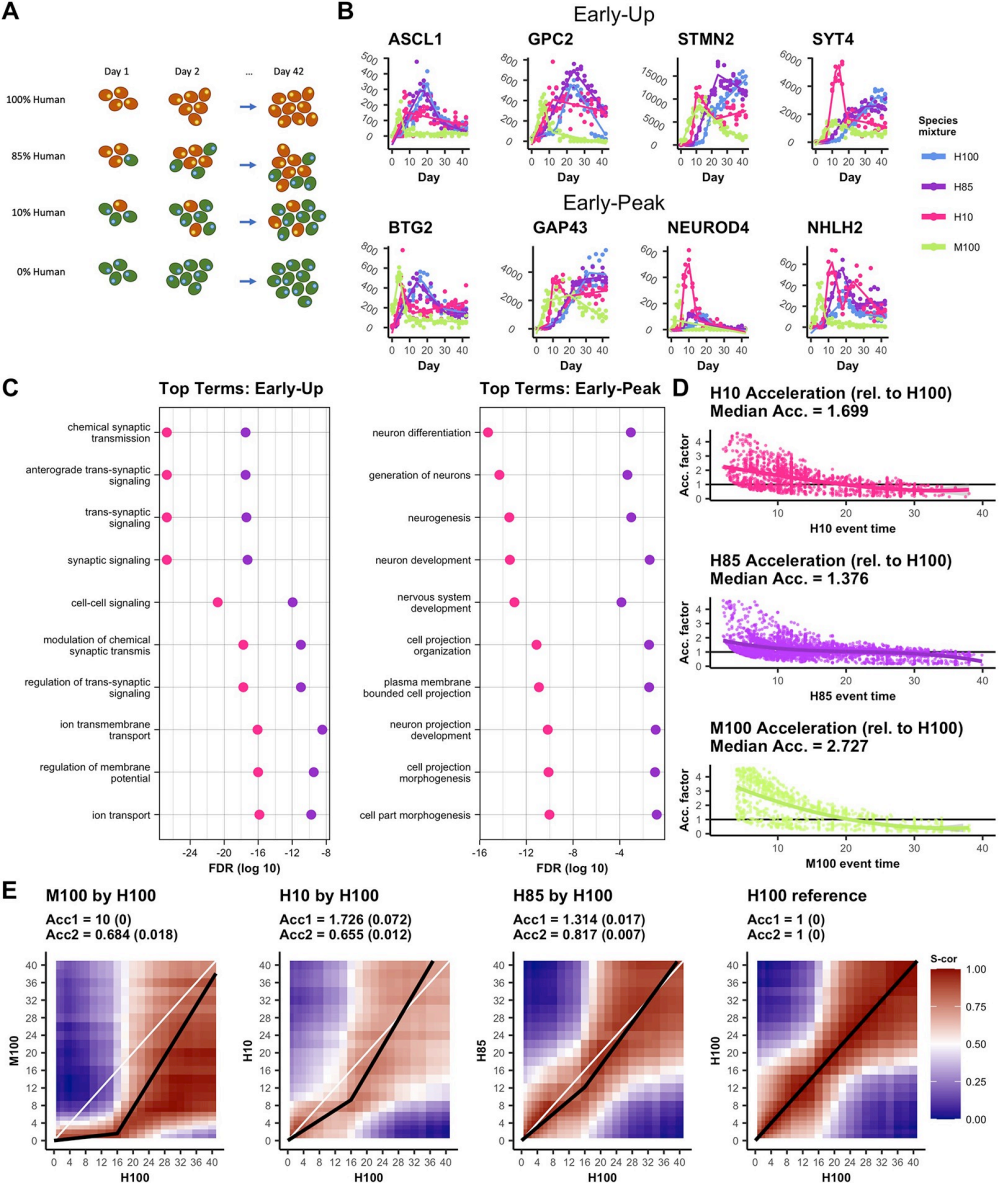
Variable mixing proportions show a dose response of acceleration effects. (A) An additional, intermediate interspecies mixing proportion, H85(M15), was compared to H0(M100), H10, and H100 time courses. (B) Expression plots of curated EU and EP genes with fitted trend lines (solid) for H100 (blue), H85 (purple), H10 (red), and M100 (green). Observed, normalized data are also plotted (dots). (C) Top 10 EU and EP GO terms from H10 showing relative significance of term enrichment for H10 and H85. (D) Smoothed acceleration factors are calculated between each of H10, H85, and M100 (human orthologous genes) against H100 using the method in Fig 3G (Materials and Methods). The median fitted acceleration from the first 16 days is reported. (E) Correlation (Spearman) heat maps where regions of high correlation (red) below the diagonal indicate accelerated activity where later days in H100 are correlated with earlier days in the comparison mixture. Correlations are calculated on a subset of highly dynamic genes (see Materials and Methods).

If the acceleration of hES cell differentiation was indeed mouse cell-induced, we reasoned that the rate of acceleration would be dose-dependent on the amount of mouse cells present in human co-cultures. Harnessing the data from multiple initial interspecies mixing proportions (0%, 10%, 85%, and 100% human vs mouse), we tested the dependence of the initial mixing proportion on the acceleration rate observed (Fig 4A).

Overall, expression profiles of a selection of key neuronal genes with either early up-regulation or early peaks in H85 samples were chronologically intermediate between H10 and H100 expression profiles (Fig 4B). Overlaying these trends with the expression profiles of orthologous genes in the M100 sample reveals progressively later onsets of gene up-regulation/peaks with decreasing proportions of mouse cells among these genes (Fig 4B).

To determine whether these results extended to the broader set of neuron-associated genes, we replicated the GO-term enrichment analysis in the H85 sample. Testing term enrichment on those genes which either upregulated or peaked earlier in H85 relative to H100 resulted in a list of the most significant terms with the same patterns as in H10. However, comparing term significance levels between the top 10 most significant terms in the H10 analysis and their H85 counterparts shows that, while the H85 terms were still highly significant, they were less so than the H10 terms (Fig 4C). Further, direct computation of acceleration factors of the first 16 days, based on differences in shared peak times and the starts of up trends, resulted in progressively decreasing calculated accelerations with stepwise drops in percentage mouse cells: 2.727 (H0/M100), 1.699 (H10/M90), and 1.376 (H85/M15), consistent with a dose-response effect on acceleration (Fig 4D).

Pairwise correlations allowed us to further aggregate relative expression trends across genes. We took a subset of genes, targeting those with dynamic expression over time, and plotted correlations calculated between pairs of time points relative to H100 (Fig 4E). Mouse orthologs demonstrate a visually significant acceleration with day 2 expression being highly correlated with H100 out to day 16. The H10 and H85 time courses both showed visual acceleration with regions of high correlation below the diagonal, but with respectively lower magnitudes as the proportion of mouse cells decreases. Adapting a technique for estimating acceleration factors from these correlation plots described in Rayon et al.[32] (see Materials and Methods) allowed us to compute average acceleration over the first 16 days independently of peaks or other expression events. We observed similar acceleration dynamics with correlation-based acceleration factors of 1.726 over the first 16 days for H10 and 1.314 over the first 16 days for H85 (Fig 4E). These correlation results, while dependent on specific geometries of the correlation plots, are themselves supported by comparing to the deconvolution of H10, H85, and H100, a procedure which is similarly based on a large panel of dynamic genes. In the deconvolution analysis, we observed higher proportions of mouse cells in mixtures resulting in the progressively earlier sequential maturation of progenitor and intermediate cell types (S9 Fig).

### Human stem cells co-cultured with mouse cells correlated with *in vivo* human fetal neocortical samples earlier than human cells alone

**Fig 5:**
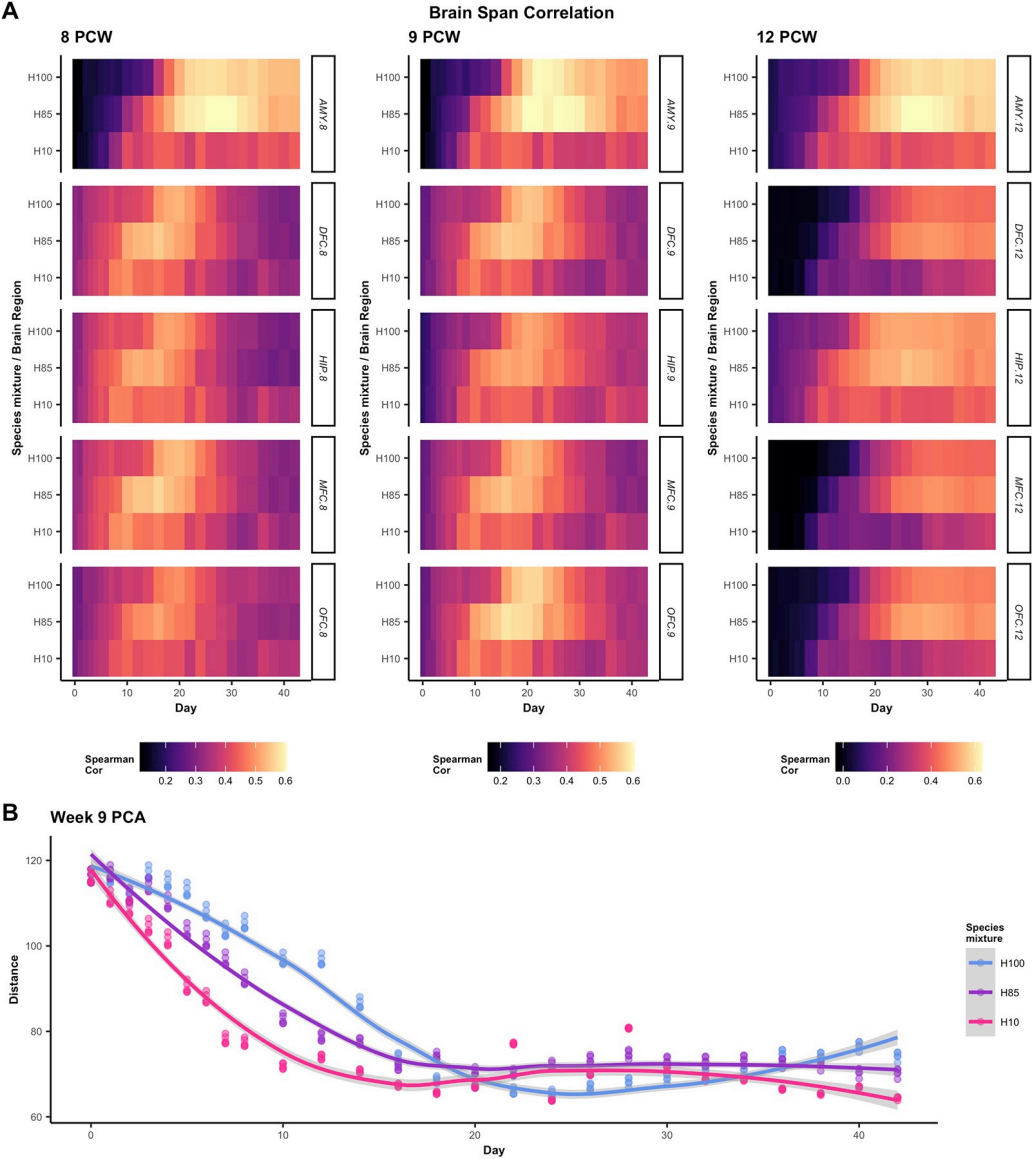
Comparison with Brain-Span regions further demonstrates a dose-response in acceleration effects. **(**A) Correlations (Spearman) between fitted trends and Brain-Span data are calculated at three Brain-Span time points and across the five brain regions represented at all three times. Calculations are performed on a subset of highly dynamic genes (see Materials and Methods). (B) Dissimilarity (PCA-based distance, see Materials and Methods) between species mixtures and each of the 5 reference brain regions are computed for each day and smoothed to estimate a continuous dissimilarity metric over time.

We compared our data with the *Brain Span* human fetal sample references to assess if our *in vitro* acceleration is consistent with sample maturity *in utero* [33–35]. We calculated correlations between our observed in vitro data and five tissue regions from the *Brain Span* database across weeks 8, 9, and 12 of development (see Materials and Methods for details). Across all time points and tissues, our mixed H10 and H85 samples increased correlation with the *Brain Span* reference earlier than the H100 control in a manner that was dose-dependent (Fig 5A).

A complementary analysis based on a variation of principal component analysis (PCA) [36,37] replicates these findings. Dimension reduction of the gene expression data allows the distance between the representation of Brain Span references and the representations of our experimental data to be interpreted as a dissimilarity metric (see Materials and Methods). Smoothing across regions for the week 9 reference, we observed that H10 minimizes dissimilarity between days 12–16, which is before H85 (days 16–20), which is further before H100 (days 20–24) (Fig 5B). Accelerated correlation to *in vivo* data was also confirmed through a similar analysis of annotated brain tissue from the *Human Protein Atlas* [38,39] (S10 Fig), consistent with a genome-wide neural program that is activated earliest in M100, then significantly accelerated in H10, followed by moderately earlier in H85, and latest in H100 samples.

While we leave the determination of mechanisms responsible for regulating the developmental clock to future work, comparisons of accelerated genes with curated gene sets allowed us to speculate on candidate pathways and transcription factors/miRNAs that may be involved [40–48]. Enrichment of the differences between up-trend/peak times (see Materials and Methods) identified signaling pathways activated earlier in both H10 and in M100 compared to H100 samples, including G-protein coupled receptor (GPCR) signaling pathways and miRNA-regulated pathways MAPK/ERK (MIR4801, MIR4731) [49,50], and PI3K/AKT [51,52], which may play roles driving developmental rates (S11 Fig and S3 Data). We also identified developmental regulators of interest, such as *NSRF*, a master neural developmental regulator essential for gastrulation that may also influence the expression of thousands of genes during development [53–56] and *OCT1*, an essential regulator of development that plays crucial roles in the earliest cell fate decisions during embryonic development [57–59], that may warrant further investigation.

## Discussion

In this study, we report for the first time multifaceted effects of interspecies mixing on the differentiation of hES cells. Through comprehensive RNA-seq time courses, we uncover that co-differentiation of hES cells intermixed with mEpiS cells was sufficient to accelerate components of neural gene regulatory programs, and identified genes with roles in neural lineage and regional identities that were both temporally and differentially expressed. We went on to demonstrate that the acceleration effect was dose-dependent on the starting ratio of interspecies cells (Fig 4), and that the chimeric samples correlated to *in vivo* tissue samples earlier in the differentiation time course than human samples alone (Figs 5 and S10).

Previously, we reported that the faster differentiation of mouse cells compared to human cells may be in part caused by increased speed of transcriptional upregulation of genes, indicated by steeper slopes in gene expression over time [60]. Consistent with a mouse cell-induced acceleration of human cell neural differentiation, here we found that the slopes of peaked genes in human cells co-differentiated with mouse cells were also significantly increased in accelerated genes compared to control samples (S5B Fig). However, non-peaking, mostly monotonic, genes whose upregulation began earlier showed lesser slopes in chimeric samples, despite starting their upward trend significantly earlier and often continuing upwards for the duration of the time course (S5A Fig). These results may suggest different functional roles of early-upregulated monotonic genes compared to genes with peak expression profiles. Indeed, genes with increased slopes and earlier peaks were significantly enriched in processes of generation of neurons and neuron cell projections, whereas earlier upregulated monotonic gene trends with lesser slopes were enriched in neuron and synaptic signaling events (Fig 3). Although we identify differences in gene expression profiles in our time course in this report, the functional maturity of resulting neurons in control versus chimeric co-differentiation conditions remains to be determined.

The mechanisms regulating developmental tempos and how interspecies co-culture might affect the differentiation speed of another species remain unknown. Although cells from different species exhibit different cell cycle rates, and counting rounds of cell division has been proposed as a possible mechanism for a cell’s ability to track developmental time [61], multiple reports also suggest that cell division is not required for differentiation in a number of systems[62–64]. Cell size is also unlikely to regulate developmental speeds as many cell types are of similar sizes across species with drastically different developmental rates [65]. Another intriguing possibility is that metabolic rates, sometimes related to cell size, cell cycle, and mammalian body mass [65], could directly modulate species-specific developmental timing [66–68]. However, when removed from the body and placed into tissue culture, cells from different species exhibit similar metabolic rates, indicating variable metabolic rates are unlikely to account for the species-specific developmental speeds retained *in vitro* [69,70]. Genome size similarly does not seem well-correlated to developmental time across mammalian species [71,72].

Recently, elegant *in vitro* models of mouse and human segmentation clocks with species-specific timing have been reported and are being used to study factors affecting developmental time [73–76]. Two recent papers have identified a correlation between some biochemical reaction rates (e.g. protein stability and turnover rates) and developmental tempos [32,77], although if, or to what extent, intrinsic developmental clocks could be altered was not determined [77]. Here, we show that cell-cell signaling alone is sufficient to affect the developmental clock. Further, we identified candidate signaling pathways and regulators activated earlier in both H10 and in M100 compared to H100 samples that may warrant future investigations (S11 Fig and S3 Data).

Previously, several studies suggested that the intrinsic species-specific developmental timer was faithfully retained under various conditions, including 2D vs 3D culture methods [6,7,78–80] and interspecies transplant/implantation studies into adult hosts [1,3,4,7]. While these studies revealed that non-embryonic interspecies conditions were insufficient to alter developmental time, in this study we demonstrate that factors actively driving an embryonic developmental program from pluripotency, rather than a mature host environment, can be sufficient to affect components of the developmental clock of cells from another species.

The ability of stem cells of different species to resolve conflicting developmental speeds has significant implications in the development of chimeric embryos for human organ formation [81]. With a widespread shortage of immunologically-matched organs for patients in need of organ transplants, the ability to grow transplantable human organs through human stem cell chimeric contributions to embryos remains an interesting potential therapeutic approach [82,83]. However, many barriers remain, including poor human chimeric contributions, possibly in part due to the vastly different developmental rates between neighboring cells of different species [8,81,84]. In this study, we demonstrate that it is possible for mouse cells to influence developmental rates and outcomes of neighboring human cells.

Previous reports of successful human cell contributions to chimeric mammalian embryos [82,85,86], including a recent report of the highest contribution (4%) of human cells in mouse-human chimeric embryos [87], could imply that human pluripotent stem cells may be induced to accelerate their developmental rate to match that of their embryonic host species. However, maturation rates of human cells in interspecies chimeras have not been well characterized. Our comprehensive time course results in this study indicate that human developmental time could be accelerated by co-differentiating cells within chimeric embryos, although collateral impacts in cell lineage outcomes may occur. In the case of neural differentiation in this study, we did find genes involved in dorsal forebrain development, for example, that were temporally downregulated in interspecies samples while genes involved in ventral midbrain development were upregulated, likely, at least in part, due to an earlier and increased exposure to *Shh* (Figs 3–5) [88–90]. Importantly, mouse and human brains do not share identical brain physiologies, cell type compositions, nor brain region proportions [91,92], so it is perhaps not surprising that some altered cell fate choices are made when cells are exposed to signals intended to created divergent outcomes. Thus, it will be important to monitor cell outcomes in chimeric embryos for human organ growth to verify that cell type contributions and organ functions are not affected.

Although the protocol described here will not have clinical applications due to the xenogenic nature of the conditions, it does suggest that the human developmental clock can be accelerated. Although the specific factors involved and clock mechanism itself remain to be dissected, this proof-of-concept report provides evidence that the species-specific developmental clock may be amenable to acceleration for clinically-relevant benefit.

## Materials and methods

### Ethics statement

All experiments described in this study were approved by the ethics committee with IRB Approval Number: SC-2015-0010. The H1 hES cells are registered in the NIH Human Embryonic Stem Cell Registry with the Approval Number NIHhESC-10-0043.

### Cell culture

Human ES and mEpiS cells were cultured and passaged as previously reported[1]. Briefly, H9 cells were cultured in E8 Medium (Thermo Fisher Scientific, USA) on Matrigel-coated plates and split every 2–3 days with EDTA. To easily identify human from mouse cells, H9 cells were electroporated with a selectable PiggyBAC-inserted plasmid expressing nuclear-localized H2B-mCherry driven by the EF1α promoter, and clonally expanded.

EGFP-expressing mEpiS cells derived from C57BL/6-Tg(CAG-EGFP)1Osb/J (JAX Stock No. 003291) mice and cultured as previously described[1,16,18]. Cell were maintained on low passage MEFs and cultured in DMEM/F12 medium (Thermo Fisher Scientific, USA) supplemented with 20% Knockout serum replacement (Thermo Fisher Scientific, USA), 0,18 mM B-mercaptoethanol (Sigma, USA), 1Xnon-essential amino acids (Thermo Fisher Scientific, USA), 2 mM L-glutamine (Sigma, USA), 7.5 ng/mL activin A (R&D Systems, USA), and 5ng/mL bFGF (R&D Systems, USA). Cells were passaged by adding TrypLE (Thermo Fisher Scientific, USA) and seeding onto fresh MEFs with 10 μM Y27632 ROCK inhibitor overnight to increase cell survival (Tocris Bioscience, UK).

### Neural induction and sampling for RNA-seq

At day 0 of time courses, H9-H2BmCherry and EGFP-mEpiS cells were washed with PBS (Thermo Fisher Scientific, USA), treated with TrypLE (Thermo Fisher Scientific, USA) for singularization, and resuspended in a simple neural differentiation medium consisting of DF3S (DMEM/F-12, L-ascorbic acid-2-phosphate magnesium (64 mg/L), sodium selenium (14 μg/L), and NaHCO3 (543 mg/L), Thermo Fisher Scientific, USA), 1XN2 supplement (Thermo Fisher Scientific, USA), 1XB27 supplement (Thermo Fisher Scientific, USA), and 100ng/mL of mNoggin (R&D Systems, USA). To aid cell survival, 10 μM Y27632 ROCK inhibitor (Tocris Bioscience, UK) was added on day 0, and cells were mixed at the indicate mouse-human ratios and seeded into Matrigel-coated 12-well plates at 2.5X10^5^ cells/well in triplicate. Media in all wells was replaced with fresh neural differentiation media (without ROCK inhibitor) every day for the 42 days of differentiation. When cells become over-confluent cells were split 1:3 or 1:6 by EDTA-treatment to avoid disrupting cell-cell interactions.

### Flow cytometry, microscopy, and time lapse imaging

Human-mouse cell ratios were established by monitoring red and green fluorescence, respectively, by flow cytometry. Cells were treated with 350μL TryPLE, spun down, and resuspended in 400 μL FACS buffer (PBS + 5% Bovine Serum Albumin). Cells were analyzed on a BD FACSCanto II and analyzed using FlowJo 9.3 software (Becton Dickinson & Company, USA).

For all cell sorting experiments, samples were lifted in either TrypLE (Thermo Fisher Scientific, USA) or 1 mg/mL Collagenase/Dispase (Roche), filtered using 40um cell strainers, and sorted on a BD FACSCanto II (Becton Dickinson, USA) according to red or green channels for human and mouse cells, respectively. Between 500,000–2,500,000 cells were sorted for each sample at each time point from day 0 to day 33. All samples pre- and post-sort were kept on ice or in a 4C water bath prior to lysis in 350uL or 700uL RLT plus buffer (Qiagen, USA) and kept at -80C for future RNA-seq processing.

All time-lapse microscopy was acquired on a BioStation CT automated imaging system (Nikon Instruments, Japan). Samples from all conditions were imaged at least every other day using phase-contract and fluorescence microscopy. For time-lapse movies, cells were acquired with a 10X magnifying objective every 30 minutes for 6 days of differentiation beginning at day 1 using phase-contrast, green, and red fluorescence channels. Overlaid movies were compiled with CL-Quant software (DRVision, USA) and scale bars and time stamps added using Premiere Pro (Adobe).

### Sample processing and RNA-seq pipeline

For RNA sample collection, samples were washed with 1XPBS (Thermo Fisher Scientific, USA) and lysed in 700 μL RLT-PLUS buffer (Qiagen, USA), and stored at -80C until further processing. Total RNA was then purified from 350 μL RLT-Plus Buffer using RNeasy Plus 96 and Micro Kits (Qiagen, Netherlands) and quantitated with the Quant-iT RNA Assay Kit (Thermo Fisher, USA). RNA was diluted to one hundred nanograms for input. The Ligation-Mediated Sequencing (LM-Seq) protocol was used to prepare and index all cDNA libraries (Hou et al 2015). Final cDNA libraries were quantitated with the Quant-iT PicoGreen Assay Kit (Thermo Fisher, USA). Twenty-five to forty-eight uniquely indexed samples were pooled per lane on an Illumina HiSeq 2500 with a single 51 base pair read and a 10 base pair index read.

A joint hg19/mm10 transcriptome reference was built by appending hg19 or mm10 respectively to the chromosome sequences and gene symbols. Tagging the gene symbols with the ID of the reference genome ensured easy decomposition of the resulting expression estimates into mouse and human subsets of species-specific gene expression. Mitochondrial genes were removed prior to further downstream analysis or normalization due to their inconsistent abundance across samples.

The sequencer outputs were processed using Illumina’s CASAVA-1.8.2 base calling software. Sequences were filtered and trimmed to remove low quality reads, adapters, and other sequencing artifacts. The remaining reads were aligned to the joint transcriptome using RSEM version 1.2.3 with bowtie-0.12.9 for the alignment step. After ensuring accurate mapping to the human/mouse subset of the transcriptome (see below for details), identified by the respective hg19 and mm10 tags on the gene symbol, the human and mouse subsets of expected counts were separated for individual analysis.

### Mixed species sample quality control

To assess the quality of alignment to the combined human-mouse transcriptome, misalignment rates were quantified in the H100 (pure human) and M100 (pure mouse) samples. In these cases, transcripts which align to the mouse and human subset of the transcriptome respectively represent errors of misalignment. Typical misalignment rates across samples appeared to be well controlled as the majority of H100 samples aligned less than 0.5% of transcripts to mouse genes (median ~0.35%, third quartile ~0.37%). The majority of M100 samples similarly aligned less than 1.5% of transcripts to human genes (median ~0.53%, third quartile ~1.42%) (S1 Fig).

A few samples (~5%) exhibited high misalignment rates (>5%). For this reason, samples with unusually low sequencing depth were removed. The filtering criteria considered log10 transformed sequencing depth (within sample sum of total expression) and removed samples with depth below the median minus 1.5 times the IQR. This procedure removed the majority of individual samples in H100 and M100 with high alignment error rates. Therefore, misalignment is believed to be primarily a function of, or at least well predicted by, low sequencing depth (S1 Fig).

A second filter was implemented to remove samples with expression profiles significantly different from biological replicates of the same time point and temporally neighboring samples. Normalized data (see below for details) from the top 1000 highest variance genes across samples within each mixture was reduced to 10 principal components. This number roughly accounts for the majority of temporal variability based on the variance explained by each component. Loadings for each component were expected to follow a smooth curve in time, following the portion of the developmental trajectory defined by the principal component. For this reason, loadings were fitted with a 4^th^ degree spline regressed against time. Studentized residuals were tested for being significantly different than the regression curve. A sample level p-value was derived by testing against the null distribution that the maximum residual across the 10 components (in absolute value) was t-distributed. The method of Benjamini and Hochberg[93] was used to provide adjusted p-values. A backward elimination and forward selection procedure was then applied. Specifically, the sample with the smallest adjusted p-value below 1e-05 was removed and the process repeated until no samples had an adjusted p-value below 1e-05 (if a sample is the last remaining observation from a particular time point, it was not considered for removal regardless of its adjusted p-value). Samples were then added back in one-at-a-time in the order of removal. Any with adjusted p-values above 1e-05 were retained for further analysis, and otherwise were rejected permanently. The filtered dataset was renormalized prior to analysis.

Empirically, this procedure was shown to remove several remaining high-error samples from M100 without removing high sequencing depth samples across species mixture groups (S1 Fig).

### Normalization of mixed species samples

We used a modified application of the scran[94] method for normalization of the expected count data. Human and mouse aligned transcripts were normalized separately, and so relative levels of normalized expression were not directly comparable between species. Consider the human mixtures (H10, H85, or H100); mouse mixtures were normalized identically. When biological replicates existed for a time point, scran was first applied to normalize these samples. Average normalized expression of biological replicates was then normalized, again via scran, across both time points and mixtures.

### Segmented regression and gene-trend classification

The dynamics of gene expression through time were defined by a segmented regression implemented using the Trendy[24] package. Trendy automatically selects the optimal number of segments (up to a maximum of 5 in this application) and requires that each segment contain a minimum number of samples (5 in this application). Additionally, an automatic significance test on segment slopes classifies segments as increasing, decreasing, or flat. As the test is itself somewhat conservative, we used a significance threshold of 0.1 (default) to determine these slope classifications. Trendy was then applied to all genes for which the 80% quantile of normalized expression is above 20 for at least one mixture.

Following regression, the segment trend classifications were used to define sets of genes by patterns of behavior relative to a reference dataset (H100 in the majority of the published analysis). Genes were classified into subsets of accelerated or differentially expressed (DE) relative to the reference dataset according to the following criteria:

1. Accelerated by Early Up (EU):
  - Both the test gene and the reference gene contain an increasing segment which is not preceded by a decreasing segment. If multiple such segments exist, only the first is considered.
  - The increasing segment in the test gene must start at least 2 days before the increasing segment in the reference gene.
  - The slope of the increasing segment in the test gene must be at least 5 times the slope of the (non-increasing) reference segment which contains the start time of the test increasing segment (typically the segment just prior to the increasing reference segment). This filter removes genes for which the reference segment containing the start time is labeled as flat by Trendy (slope is not significantly different from 0), but is fitted with an up-trending slope. This can happen in instances where the reference segment is short and so does not contain enough sample points for the up-trend to be labeled as significant.
2. Accelerated by Early Peak (EP):
  - Both the test gene and the reference gene contain a peak defined by an increasing segment followed by a flat or decreasing segment. The peak itself is defined by the time of the breakpoint between these two segments.
  - The peak in the test gene must be at least 2 days before the peak in the reference gene.
3. DE Up:
  - The maximum fitted value of the test gene plus 1 must be at least 3 times the maximum fitted value of the reference gene plus 1. The inclusion of the plus 1 bias to each side prevents very lowly expressing genes from appearing DE due to small differences in fitted values which are only multiplicatively large due to the low overall expression.

Genes in H10 or H85 matching these acceleration/up-regulation criteria were denoted as “Early” or “Up” respectively.

We also ran this classification denoting H100 as the test datasets. When genes matched the criteria in this case, we denoted the corresponding gene in the reference dataset, H10 or H85, “Late” or “Down” according to the specific criteria met.

### Acceleration factor estimation

Point estimates of the relative acceleration of one dataset compared to another were computed from genes which either peak in both datasets or trend up in both datasets. For simplicity, consider the case of H10 relative to H100. From peaking/up-trending genes, the event time was calculated: time of peak or time of the start of up-trend respectively. When a gene both peaks and trends up, the peak was preferred as it was assumed to be a more accurate estimate of regulatory changes. Up-trending genes (without peaks) which start up-trending in either H10 or H100 on day 0 were discarded. Point estimates were then calculated as the ratio of the event time in H100 to the event time in H10. In this way, a ratio of 2 would indicate that, at the time of the even in H10, that gene is accelerated to be 2x as fast as the gene in H100. Point estimates are computed across pairs of datasets.

To compute a continuous estimate of acceleration factors, the above point estimates are smoothed using spline regression (linear model in R with a basis spline under default parameters) against event time in the test (e.g., H10) dataset. It should be noted that these acceleration factors are best interpreted as estimates of the relative acceleration of the genes which are active at that time point. Acceleration factors of 1 therefore identify time points, and thereby sets of genes active at that time point, which are relatively unchanged between the conditions.

### Gene set enrichment

Accelerated and DE gene sets were further characterized through testing for GO term enrichment. The topGO[95] package and org.Hs.eg.db[96] dataset were used to perform enrichment testing on GO terms belonging to the biological processes (BP) ontology. The set of all genes on which Trendy segmented regression was run was used as the background set (see above for subset definition). Significant p-values were then FDR corrected[93] prior to analysis.

Pathway and transcription factor/miRNA enrichment was performed in a similar manner. In these cases, the piano[97] package was used to accommodate non-binary statistics. Specifically, enrichment was performed on the difference between up-trend or peak events between a test dataset (e.g., H10) and a reference dataset (e.g., H100). When available, the difference was calculated from the time of peaks in each dataset. Absent peaks, the difference was calculated from the time of the start of up-trends. Genes without either shared peaks or shared up-trends were given a difference of 0.

Enrichment for these differences were performed against two collections of gene sets from the MSigDB database[40,41]. The first was a curated collection of pathways, including KEGG[42–44], Biocarta[45], and Reactome[46] sets of gene pathways. The second was a collection of miRNAs[47] and transcription factors[48] (TFs) and downstream regulated genes. Enrichment was performed with the runGSA function from the piano package (4e6 permutations, minimum gene set size of 1, maximum gene set size of 250).

### Sorted sample quality control validation

Sorted samples, sH100, sH10, sM90, and sM100 were similarly aligned to a combined transcriptome (as described above) to provide a validation dataset. One data point was removed for low sequencing depth (day 29 from sH10, fewer than 1e3 expected counts where typical sorted samples had greater than 1e6 expected counts) and all others were retained.

Empirical misalignment rates were computed for sH100 and sH10 as the fraction of expected counts aligned to the mouse portion of the transcriptome; median values across days were 0.53% and 2.23% respectively.

Active misaligned genes were identified as genes in the off-target portion of the reference transcriptome (e.g., mouse genes for sH10) with an 80% quantile of expected counts ≥ 20. Enrichment following the above-described procedure was performed on these gene sets.

Normalization was performed using the calculateSumFactors function in scran (default parameters) to compute scale factors which expected counts were then divided by. As with the other data, Trendy was used to perform segmented regression (maximum 4 breakpoints, minimum 2 points per segment, p-value threshold 0.1). Output from Trendy was used to classify genes as EU/LU. Peak analysis was omitted as the lower resolution of the data prevent robust identification of peaks (e.g., visually identifiable peaks are not significant under Trendy regression). Acceleration factor estimation was computed from these shared up-trend genes in the above-described manner, and enrichment was performed on EU genes, again as above.

### Correlation analysis

Expression similarity across time points, species mixtures, and external reference datasets was assessed through gene expression correlations. To ensure that computed correlations were representative of the temporal gene dynamics being studied, correlations were computed on only a subset of genes. Highly dynamic genes were subset from all Trendy-fit genes by calculating the coefficient of variation of fitted values. The highest CV across species mixtures was then retained as a measure of each gene’s level of temporal dynamics, and the top 2000 most dynamic (highest CV) genes were subset for analysis.

Relative similarity of species-mixtures was computed as the correlation matrix (spearman type) between time points where within-day biological replicates were averaged together to obtain a single day expression value.

Similar calculations of correlations between the species-mixture data and two outside datasets, the BrainSpan atlas of the developing human brain[34,35] and the Human protein atlas[38,39], were conducted. In these cases, the genes used to calculate correlations were the union of the top 1500 most dynamic genes from H10/H85/H100 and the top 1500 most dynamic genes (highest CV across cell-types) from the relevant in vivo reference dataset.

### Correlation-based acceleration

To use in vitro correlation heatmaps to estimate acceleration factors, we adapted a technique described in Rayon et al. 2020[32]. Specifically, we performed a version of weighted regression whereby the weights derive from the correlation values. However, as the in vitro data was observed to not have a constant acceleration factor, we performed segmented regression with a fixed breakpoint at day 16. The specific function to minimize was then:

$$\underset{\theta^{*}}{\min}\left\{\sum_{i,j}cor{\left(EC_{t_{i}},EC_{t_{j}}\right)}^{2}dist_{⊥}{\left((t_{i},t_{j})|\theta^{*}\right)}^{2}\right\}$$

Where *EC* denotes expected counts (correlation is spearman type, so normalization in unnecessary), *t*_*i*_ and *t*_*j*_ denote days in the reference and test datasets respectively (e.g., H100 and H10), and *dist*_*⊥*_*()* denotes the perpendicular distance to the current estimate of the segmented regression given regression coefficients θ* from the provided time pair (coordinates on the correlation heatmap). Minimization was conducted in R using the optim function (L-BFGS-B method, upper and lower bounds of 10 and 1/10 respectively, segmented regression fixed to pass through (0, 0), initial slopes set to 1 in each segment). Standard errors for coefficient estimates were generated by bootstrapping solutions from random samples (with replacement) of the input genes. Regression slopes then defined the desired acceleration factor up to an inversion.

### In vivo dissimilarity

Dissimilarity between in vitro data (average across biological replicates for a given day and species mixture) and in vivo references was computed from highly dynamic genes (see criteria above in Correlation analysis) using a variation of principal component analysis (PCA). To accommodate the distributional properties of these sequencing data, as well as the properties of the reference data, a variation on PCA, glmpca[36,37], which uses a negative binomial model residuals was used to perform dimension reduction to 6 dimensions (6 principal components). Dissimilarity was then computed as the distance (Euclidian) between an in vitro data point in the low dimensional space and the corresponding low dimensional representation of a reference in vivo data point. glmpca was run with the negative binomial family, fisher optimizer, penalty of 10, minimum iterations of 400, and was parameterized by size factors derived from Scran to normalize the (unnormalized) expected counts from the in vitro data and the in vivo reference.

Note that the BrainsSpan data were available as reads per kilobse million (rpkm) rather than the expected counts (EC) used in this analysis. For this reason, analysis on the BrainSpan data was conducted using fpkm from the in vitro data as the best available analog.

### Deconvolution analysis

Deconvolution analyses to estimate proportions of cell-types in the observed bulk sequencing data were performed using the music_prop function (default parameters) from the MuSiC[31] package with the CoDEx database of annotated developing brain cells[30] as reference. Within species-mixture, estimated proportions were smoothed across biological replicates and days using the DirichletReg function from the DirichletReg package[98] and a basis spline (df = 4).

### R package versions

All calculations were performed using R[99] (v3.6.2) and major packages: Trendy[24] (v1.6.4), scran[94] (v1.12.1), topGO[95] (v2.36.0), org.Hs.eg.db[96] (v3.8.2), org.Mm.eg.db[100] (v3.11.4), ggplot2[101] (v3.3.0), MuSiC[31] (v0.1.1), piano[97] (v2.4.0), DirichletReg[98] (v0.7.0), glmpca[37] (v0.2.0).

## Supporting information

S1 Data Summaries of expression characteristics for genes classified as exhibiting differential timing or expression in H10. (XLSX)

S2 Data Summaries of expression characteristics for genes classified as exhibiting differential timing or expression in H85. (XLSX)

S3 Data Gene set enrichment significance levels for pathway and TF/miRNA enrichment in H10 and M100 orthologous genes relative to H100. As enrichment is performed on timing differences in peaks/starts of early-up, enrichment results are directional, providing the significance that terms are enriched for accelerated/decelerated genes.
(XLSX)

S1 Movie 10% H9-H2BmCherry (red) cells mixed with 90% EGFP+ mouse EpiS cells (green) were seeded in neural differentiation medium and were imaged from days 1–7 every 30 mins using a BiostationCT imaging system (Nikon, Japan). Media replacement occurred approximately every 24 hours. Images with focused condensation were removed. Overlaid channels of microscopy images were compiled into the movie with CL-Quant software (DRVision, USA). Scale bars = 200 μm.
(MP4)

S2 Movie Time-lapse movie of a second field of view from the identical time course described in S1 Movie, played at 3x the frame rate. (MP4)

S1 Fig Quality control filtering removes samples with uncharacteristically low sequencing depth. (A) Observed per-sample misalignment rates for pure human (H100)/pure mouse (M100) mixtures. (B) Observed log10 total sequencing depth summed across sequences aligned to either human or mouse. Most samples removed from analysis (blue) are below the depth filtering threshold (dashed line) (see Materials and Methods). Otherwise, the M100 results suggest that the higher-depth removed samples are those with higher rates of misalignment (top/middle, right column).
(TIF)

S2 Fig Seeded human cell proportions increase over time. (A) Observed percent of human cells in H10 mixture out to 16 days. (B) FACS plots intensities used to compute relative proportions of human and mouse cells in H10 mixture.
(TIF)

S3 Fig Selected gene expression plots show characteristic differences between H100, H10, and M100. (A) Early-Up classified fitted trend lines (solid) are plotted for selected genes with overlaid normalized observed data (points). (B) Similar results are shown for selected Early-Peak classified genes (green = M100, pink = H10, blue = H100).
(TIF)

S4 Fig Enrichment of late-up (LU) and late-peak (LP) genes fail to demonstrate a pattern of neuron development-related terms. (A) Top GO terms enriched for LU genes in H10 compared to H100 with corresponding FDR corrected p-values (log 10 scale). (B) Top GO terms enriched for LP genes in H10 compared to H100 with corresponding FDR corrected p-values (log 10 scale).
(TIF)

S5 Fig Up-trends show defining shifts in H10 among EU and EP genes. (A) EU genes from each of the listed GO terms are plotted. The start of uptrends between H10 and H100 are plotted (top left) with KS testing sowing significant left shift corresponding to significantly earlier trend starts in H10. Slope ratio (ratio of H10 up-trend slope over H100 up-trend slope) densities are plotted (top right) on the log scale for top enriched GO terms with KS testing showing a significant left-shift corresponding to significantly reduced slopes in H10 among these genes. Densities of the duration of up-trends (bottom left) show significantly longer (KS test) trends for H10 (red) than H100 (blue). (B) EP genes from each of the listed GO terms are plotted. The timing of peaks are plotted (top left) with KS testing showing significant left shift corresponding to significantly earlier peaks in H10. Similar results for EP genes as the above EU genes show significantly earlier up-trend starts, significant increases in slope in H10, and reduced duration of up-trends (pink = H10, blue = H100).
(TIF)

S6 Fig Expression from sorted co-culture cells fails to show misalignment bias. (A) Empirical misalignment for sH100 and sH10 are plotted by day. (B) Misaligned genes for the sH10 and sM90 (mouse and human aligned reads respectively) are subset. Enrichment testing is performed on active genes, defined as those with 80% quantile of observed expression of at least 20 expected counts, and top terms are plotted against FDR corrected p-values (log 10 scale). (C) Expression from selected genes which are accelerated in the H10-H100 comparison are plotted for sH100, sH10, and sM100, and show similar acceleration effects in this sorted control dataset. (D) EU/LU genes are tabulated for sH10. (E) Continuous acceleration factors are calculated for sH10 and top EU enriched GO terms are plotted.
(TIF)

S7 Fig Analysis of co-cultured mouse expression suggests deceleration of mouse gene expression patterns. (A-B) Genes identified as shared up-trends (excluding those which start to trend up on day 0 in both M100 and M15) or shared peaks between M15 and M100 are classified as either early, late, or unchanged, and then tabulated. (C) Shared up-trending and peaking genes are used to estimate a continuous acceleration factor for M15 relative to M100 in an identical manner to the human data. The median acceleration factor (over the first 16 days) of 0.894 indicates a deceleration in gene activity. (D-G) Top terms enriched for EU, LU, EP, and LP genes respectively are plotted against FDR corrected p-values. Neural associated terms are either unique to the late category or are more significant in that group, suggesting a deceleration effect specific to neural genes.
(TIF)

S8 Fig Up/down regulation of genes in H10 show region specific patterns. Relative expressions of curated genes in regional/functional groups are plotted on a normalized -1 to 1 scale. Gene expression (within gene) is normalized such that the maximum difference in fitted expression (in H100 or H10) equals 1. Relative expressions are then calculated as the difference between H10 and H10 where higher H10 values tend towards 1 (red), lower H10 values tend towards -1 (blue), and equivalent values tend towards 0 (black).
(TIF)

S9 Fig Deconvolution analysis of mixed-species data supports dose-response effect. Expression data for H100, H85, and H10 respectively are deconvolved relative to the CoDEx reference dataset of annotated developing brain single cell expression. Deconvolution produces estimates of the relative proportions of reference cell-types present in the bulk data. Estimates are smoothed against time and plotted for each of H100 (top), H85 (middle), and H10 (bottom).
(TIF)

S10 Fig Correlation with Human Protein Atlas (HPA) data further demonstrates dose response behaviors. Correlations (Spearman) between fitted trends HPA data are calculated across the thirteen HPA regions. Calculations are performed on a subset of highly dynamic genes (see Materials and Methods). Dissimilarity (PCA-based distance, see Materials and Methods) between species mixtures and each of 6 HPA cell-types are computed for each day and smoothed to estimate a continuous dissimilarity metric over time.
(TIF)

S11 Fig Candidate pathways, transcription factors (TFs), and miRNAs to mediate the observed acceleration. (A) Top pathways (left) and TFs/miRNAs (right) enriched for acceleration in H10 are plotted against their FDR corrected p-values. (B) Similar analysis is performed on M100 orthologs compared to H100 expression. Prior to plotting top pathways (left) and TFs/miRNAs (right), enriched terms are subset to include only those which are also significant (FDR corrected p-value ≤ 1e-2) in the above H10 comparison.
(TIF)
