# Supplementary figures and images for: Interspecies chimeric conditions affect the developmental rate of human pluripotent stem cells
Source: PLoS Comput Biol. 2021 Mar 1;17(3):e1008778. doi: 10.1371/journal.pcbi.1008778 (PMC7951976; doi:10.1371/journal.pcbi.1008778)

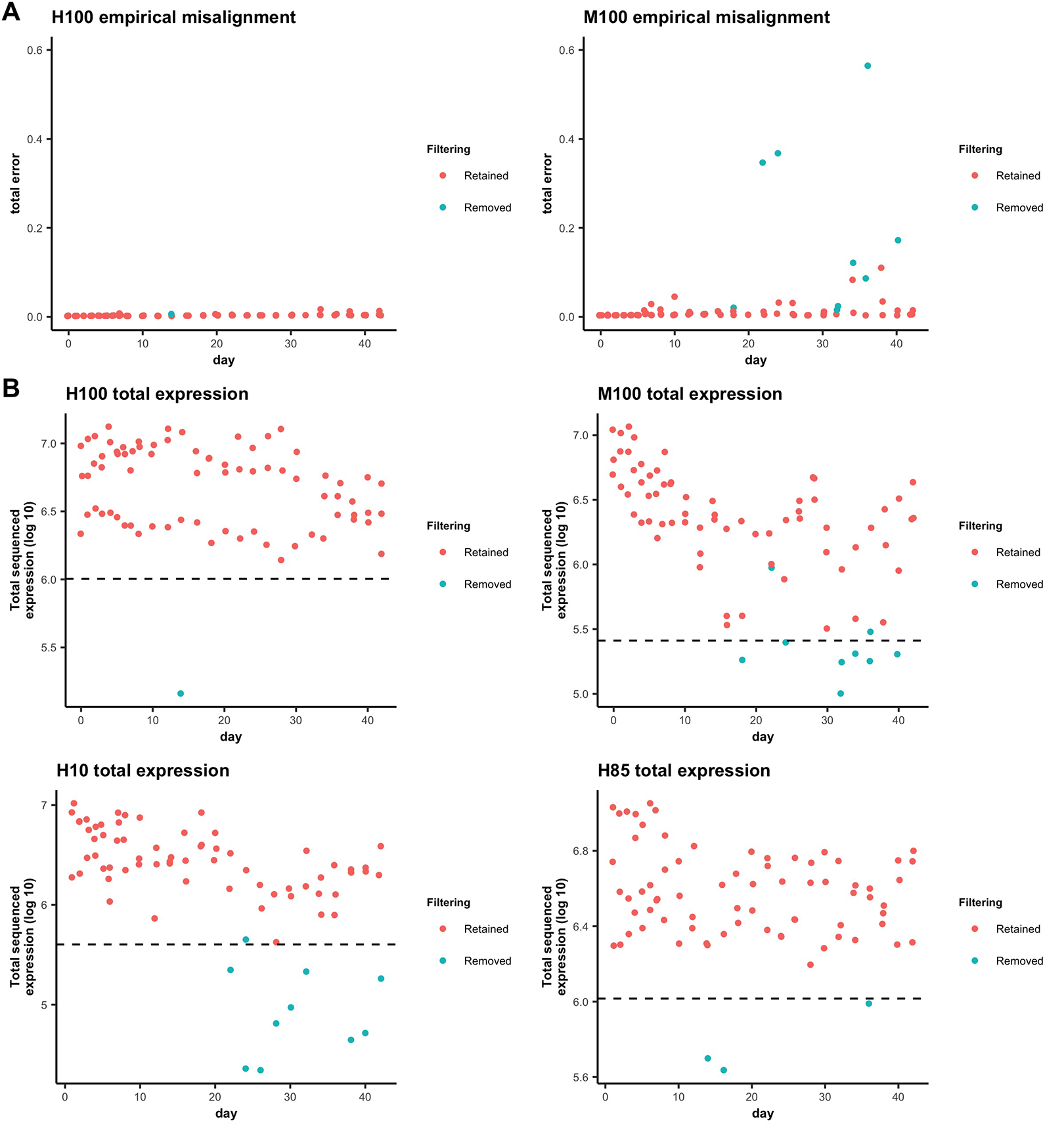

Supplement: S1 Fig — (A) Observed per-sample misalignment rates for pure human (H100)/pure mouse (M100) mixtures. (B) Observed log10 total sequencing depth summed across sequences aligned to either human or mouse. Most samples removed from analysis (blue) are below the depth filtering threshold (dashed line) (see Materials and Methods). Otherwise, the M100 results suggest that the higher-depth removed samples are those with higher rates of misalignment (top/middle, right column). (TIF) [file pcbi.1008778.s006.tif]

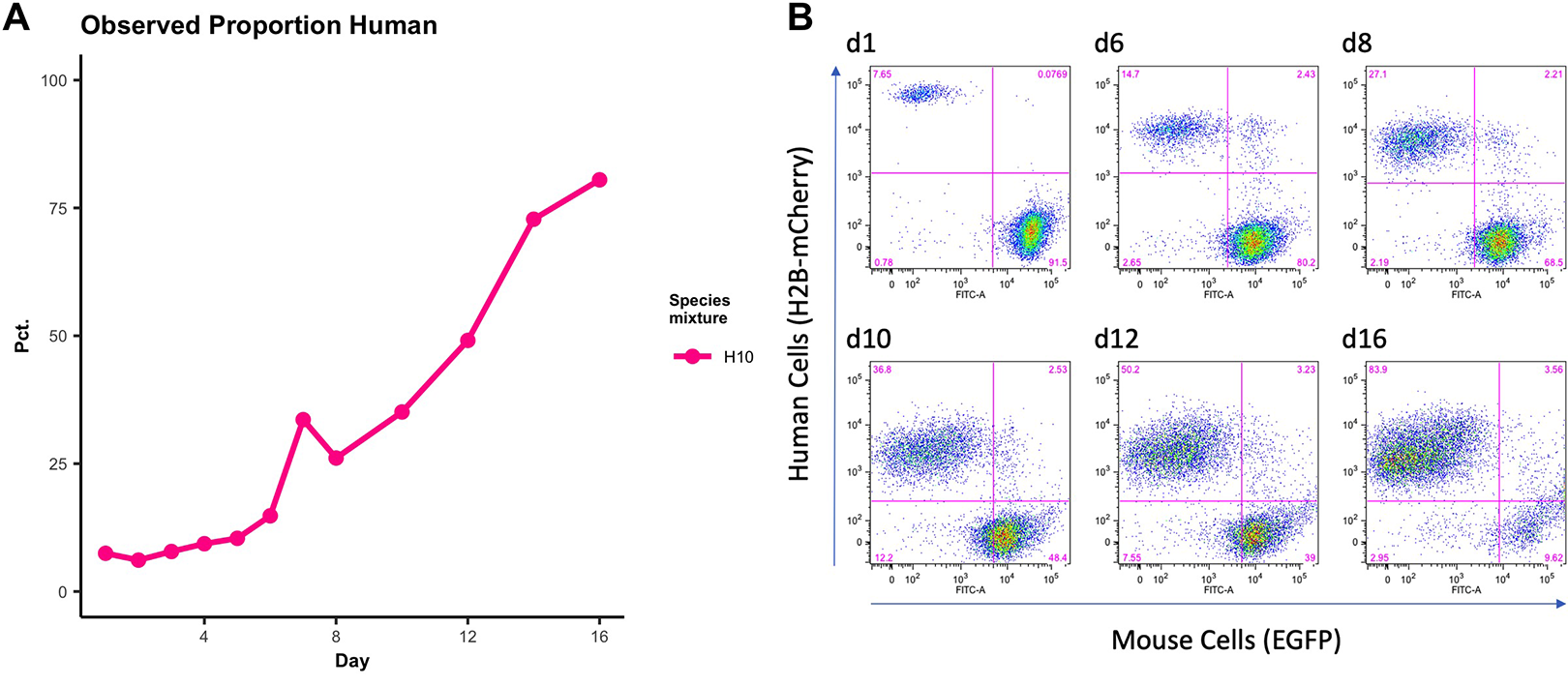

Supplement: S2 Fig — (A) Observed percent of human cells in H10 mixture out to 16 days. (B) FACS plots intensities used to compute relative proportions of human and mouse cells in H10 mixture. (TIF) [file pcbi.1008778.s007.tif]

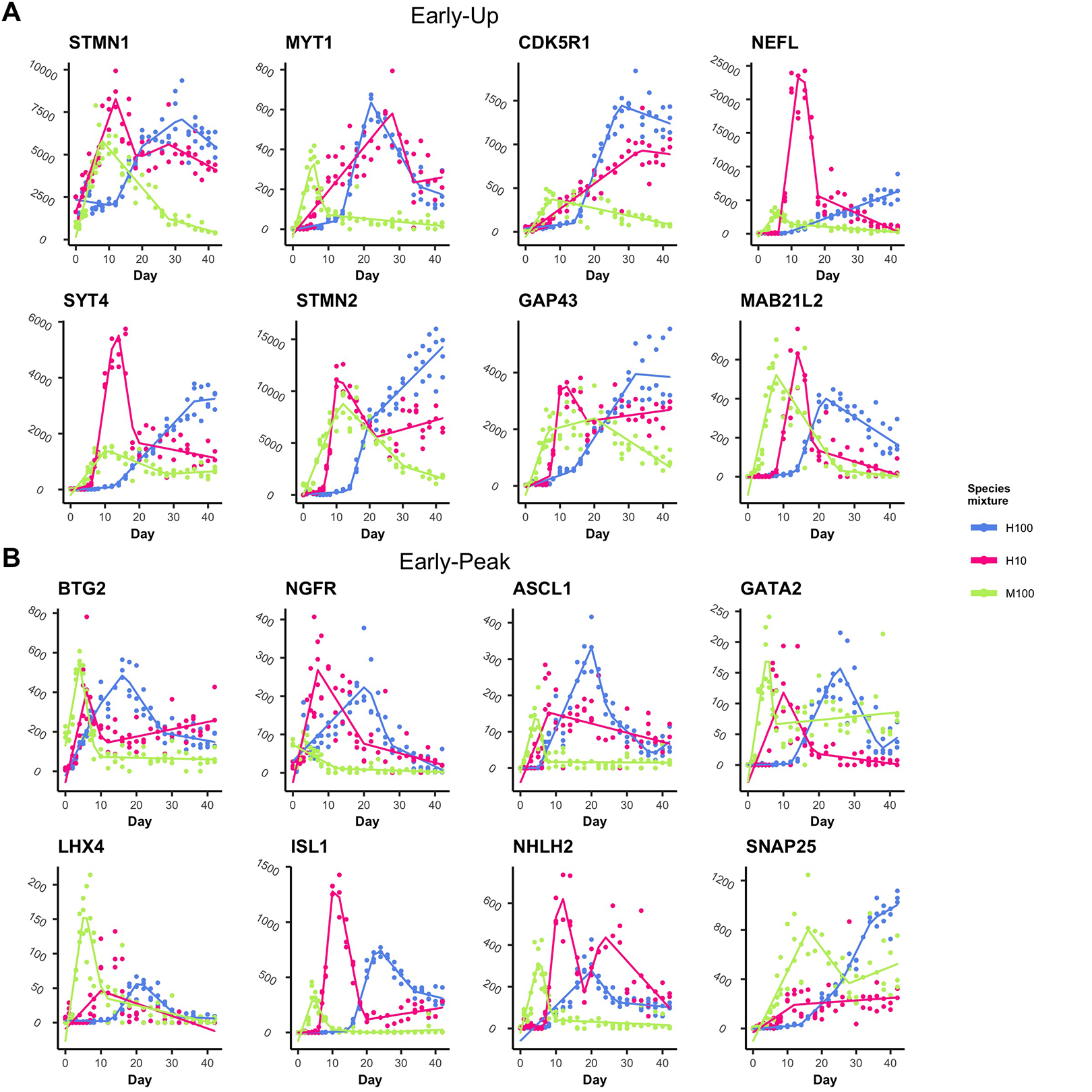

Supplement: S3 Fig — (A) Early-Up classified fitted trend lines (solid) are plotted for selected genes with overlaid normalized observed data (points). (B) Similar results are shown for selected Early-Peak classified genes (green = M100, pink = H10, blue = H100). (TIF) [file pcbi.1008778.s008.tif]

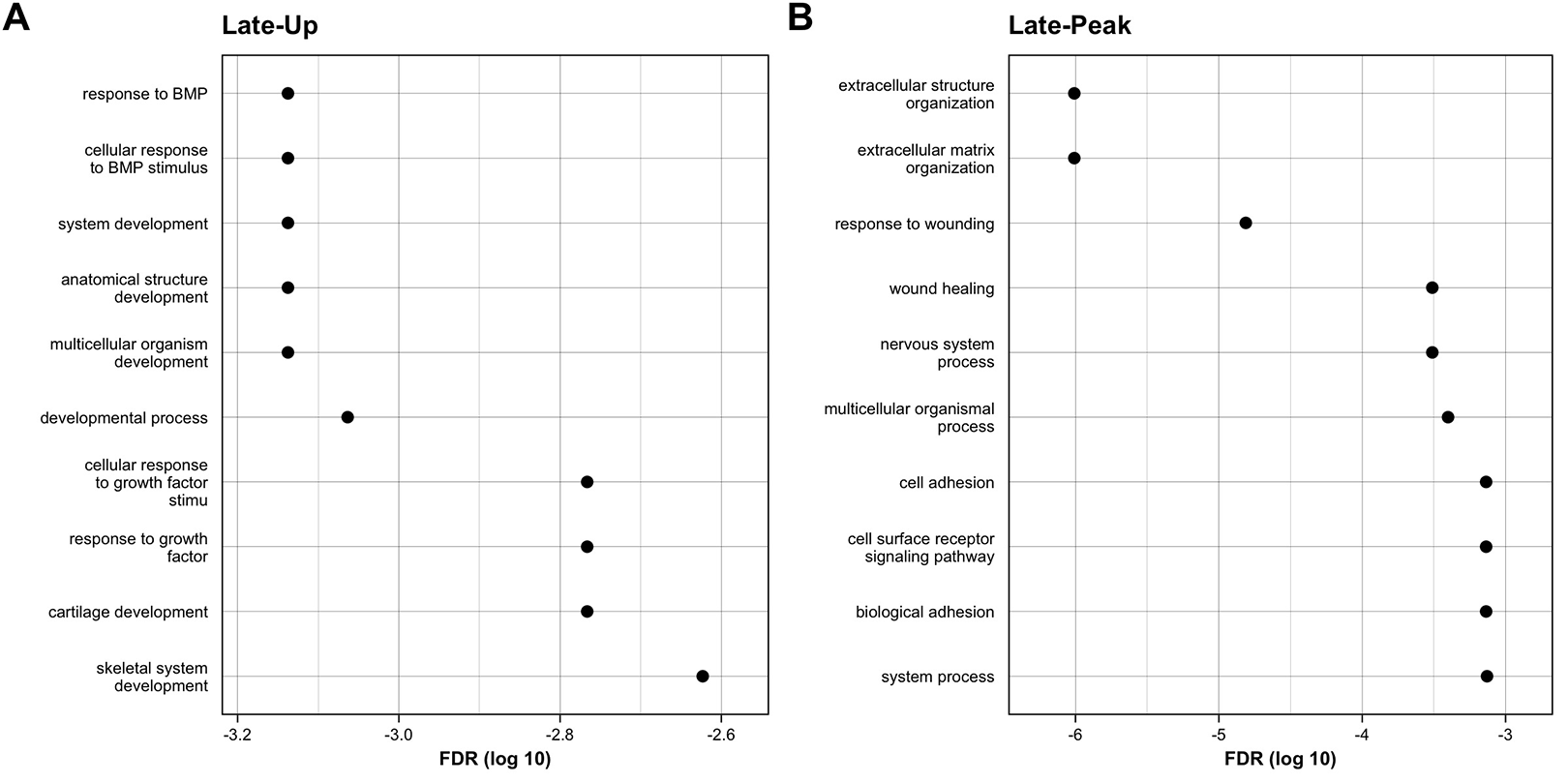

Supplement: S4 Fig — (A) Top GO terms enriched for LU genes in H10 compared to H100 with corresponding FDR corrected p-values (log 10 scale). (B) Top GO terms enriched for LP genes in H10 compared to H100 with corresponding FDR corrected p-values (log 10 scale). (TIF) [file pcbi.1008778.s009.tif]

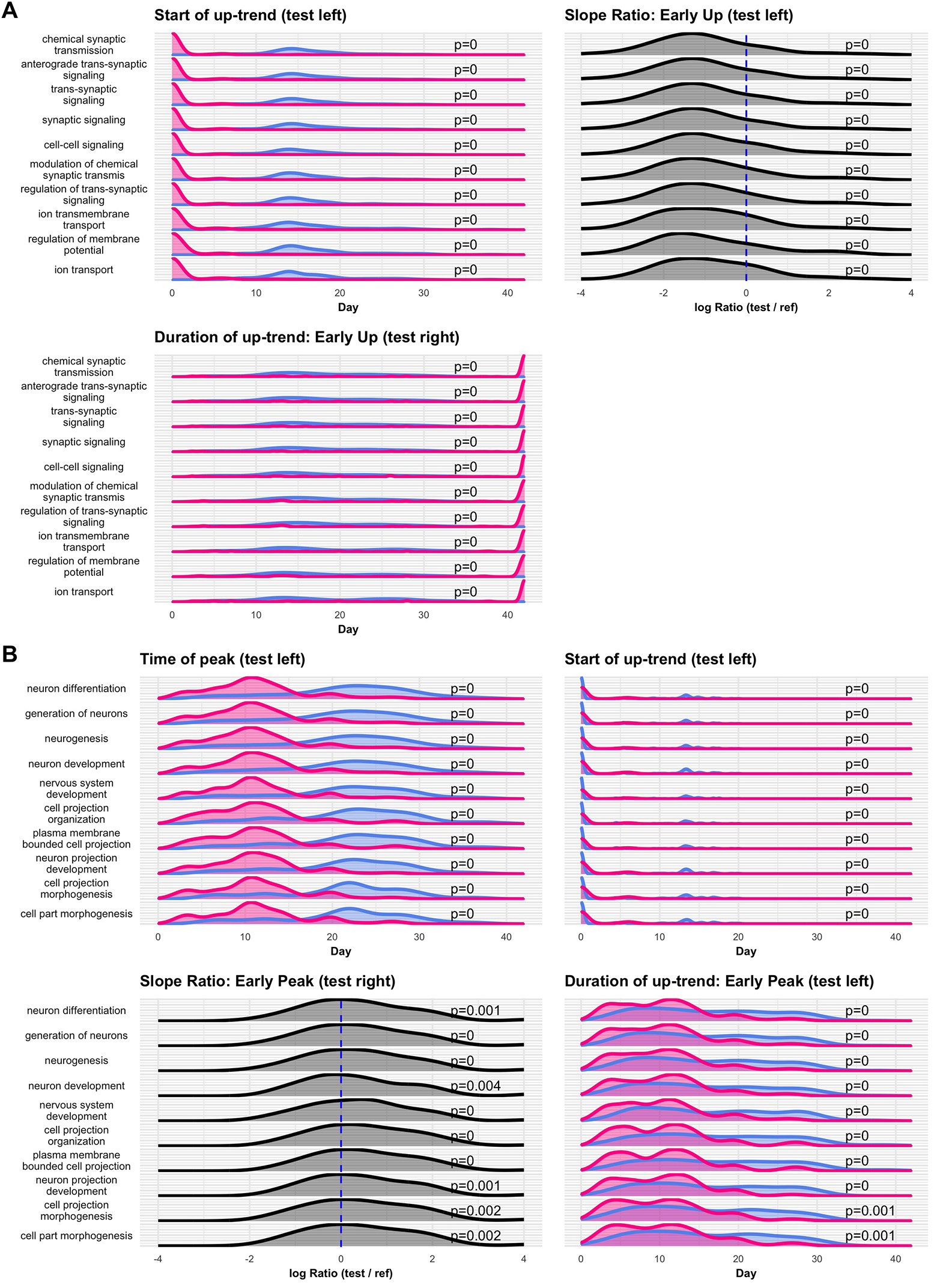

Supplement: S5 Fig — (A) EU genes from each of the listed GO terms are plotted. The start of uptrends between H10 and H100 are plotted (top left) with KS testing sowing significant left shift corresponding to significantly earlier trend starts in H10. Slope ratio (ratio of H10 up-trend slope over H100 up-trend slope) densities are plotted (top right) on the log scale for top enriched GO terms with KS testing showing a significant left-shift corresponding to significantly reduced slopes in H10 among these genes. Densities of the duration of up-trends (bottom left) show significantly longer (KS test) trends for H10 (red) than H100 (blue). (B) EP genes from each of the listed GO terms are plotted. The timing of peaks are plotted (top left) with KS testing showing significant left shift corresponding to significantly earlier peaks in H10. Similar results for EP genes as the above EU genes show significantly earlier up-trend starts, significant increases in slope in H10, and reduced duration of up-trends (pink = H10, blue = H100). (TIF) [file pcbi.1008778.s010.tif]

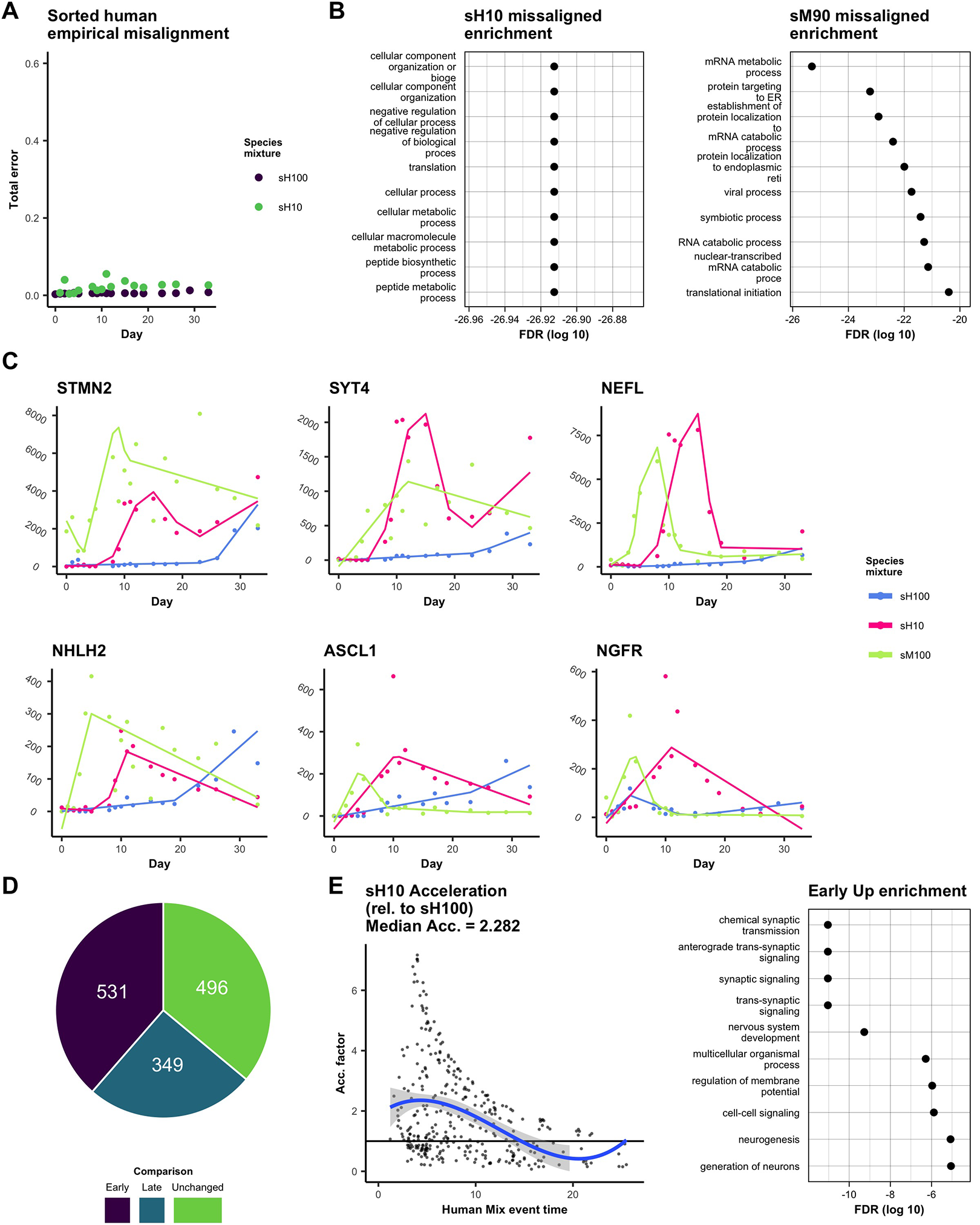

Supplement: S6 Fig — (A) Empirical misalignment for sH100 and sH10 are plotted by day. (B) Misaligned genes for the sH10 and sM90 (mouse and human aligned reads respectively) are subset. Enrichment testing is performed on active genes, defined as those with 80% quantile of observed expression of at least 20 expected counts, and top terms are plotted against FDR corrected p-values (log 10 scale). (C) Expression from selected genes which are accelerated in the H10-H100 comparison are plotted for sH100, sH10, and sM100, and show similar acceleration effects in this sorted control dataset. (D) EU/LU genes are tabulated for sH10. (E) Continuous acceleration factors are calculated for sH10 and top EU enriched GO terms are plotted. (TIF) [file pcbi.1008778.s011.tif]

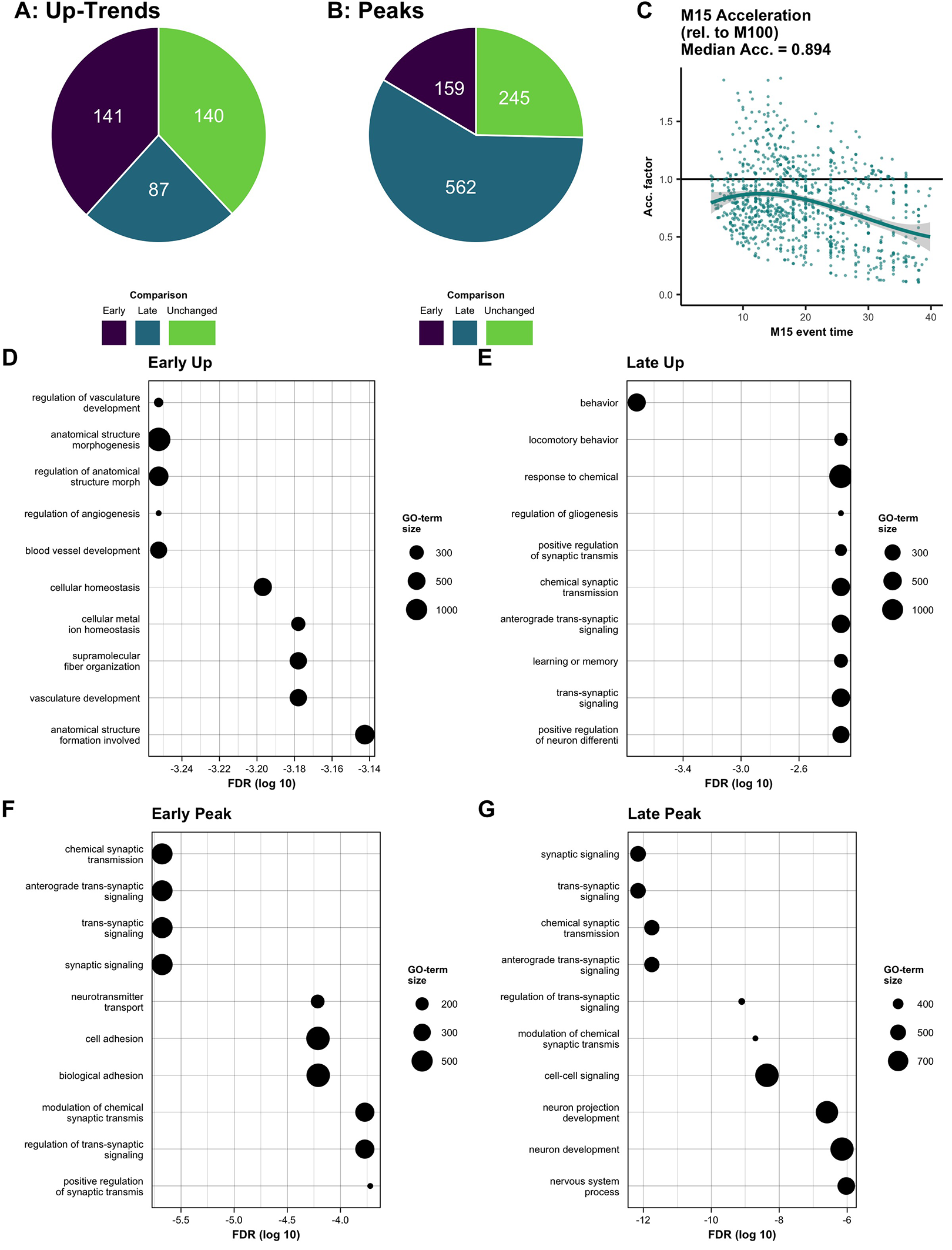

Supplement: S7 Fig — (A-B) Genes identified as shared up-trends (excluding those which start to trend up on day 0 in both M100 and M15) or shared peaks between M15 and M100 are classified as either early, late, or unchanged, and then tabulated. (C) Shared up-trending and peaking genes are used to estimate a continuous acceleration factor for M15 relative to M100 in an identical manner to the human data. The median acceleration factor (over the first 16 days) of 0.894 indicates a deceleration in gene activity. (D-G) Top terms enriched for EU, LU, EP, and LP genes respectively are plotted against FDR corrected p-values. Neural associated terms are either unique to the late category or are more significant in that group, suggesting a deceleration effect specific to neural genes. (TIF) [file pcbi.1008778.s012.tif]

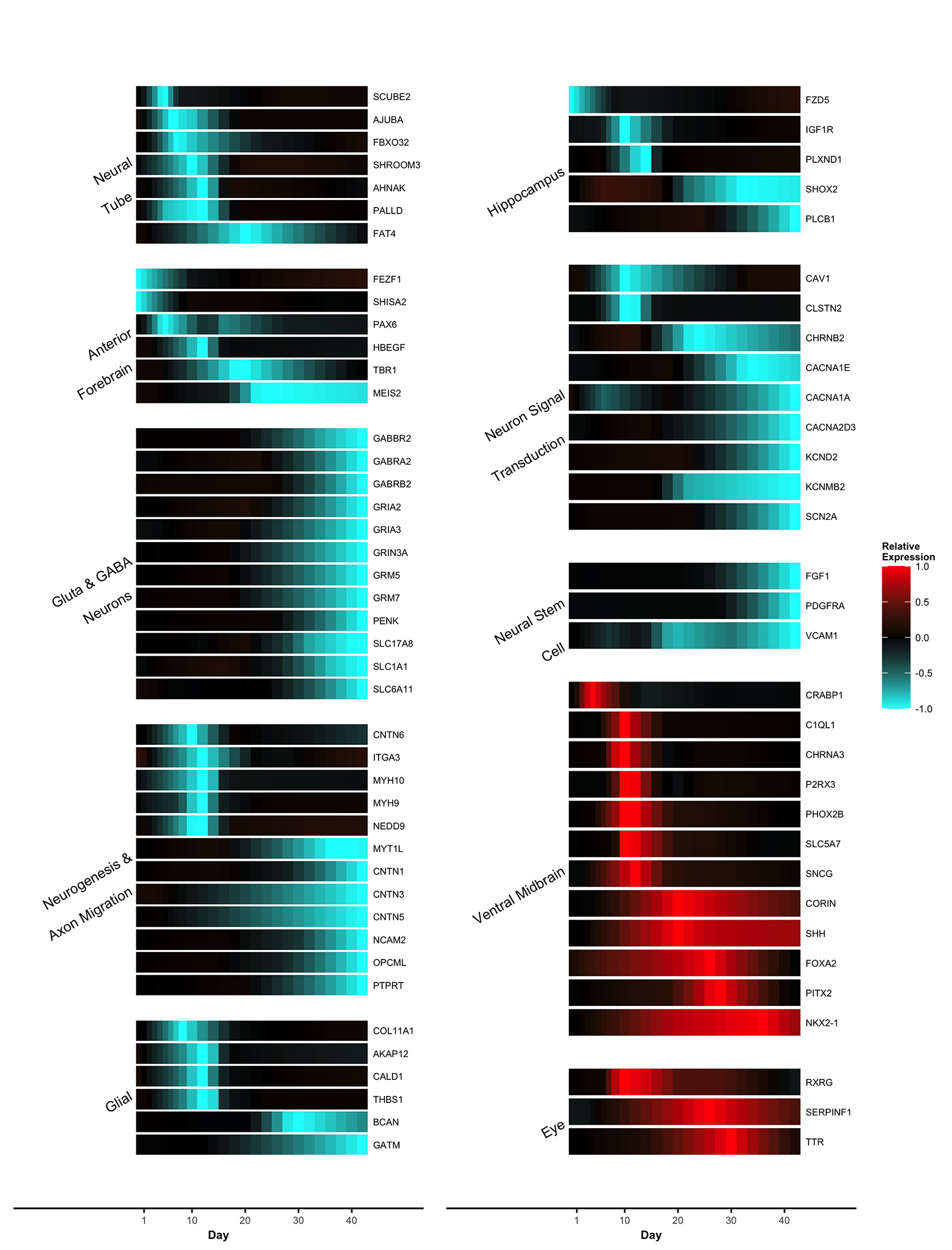

Supplement: S8 Fig — Relative expressions of curated genes in regional/functional groups are plotted on a normalized -1 to 1 scale. Gene expression (within gene) is normalized such that the maximum difference in fitted expression (in H100 or H10) equals 1. Relative expressions are then calculated as the difference between H10 and H10 where higher H10 values tend towards 1 (red), lower H10 values tend towards -1 (blue), and equivalent values tend towards 0 (black). (TIF) [file pcbi.1008778.s013.tif]

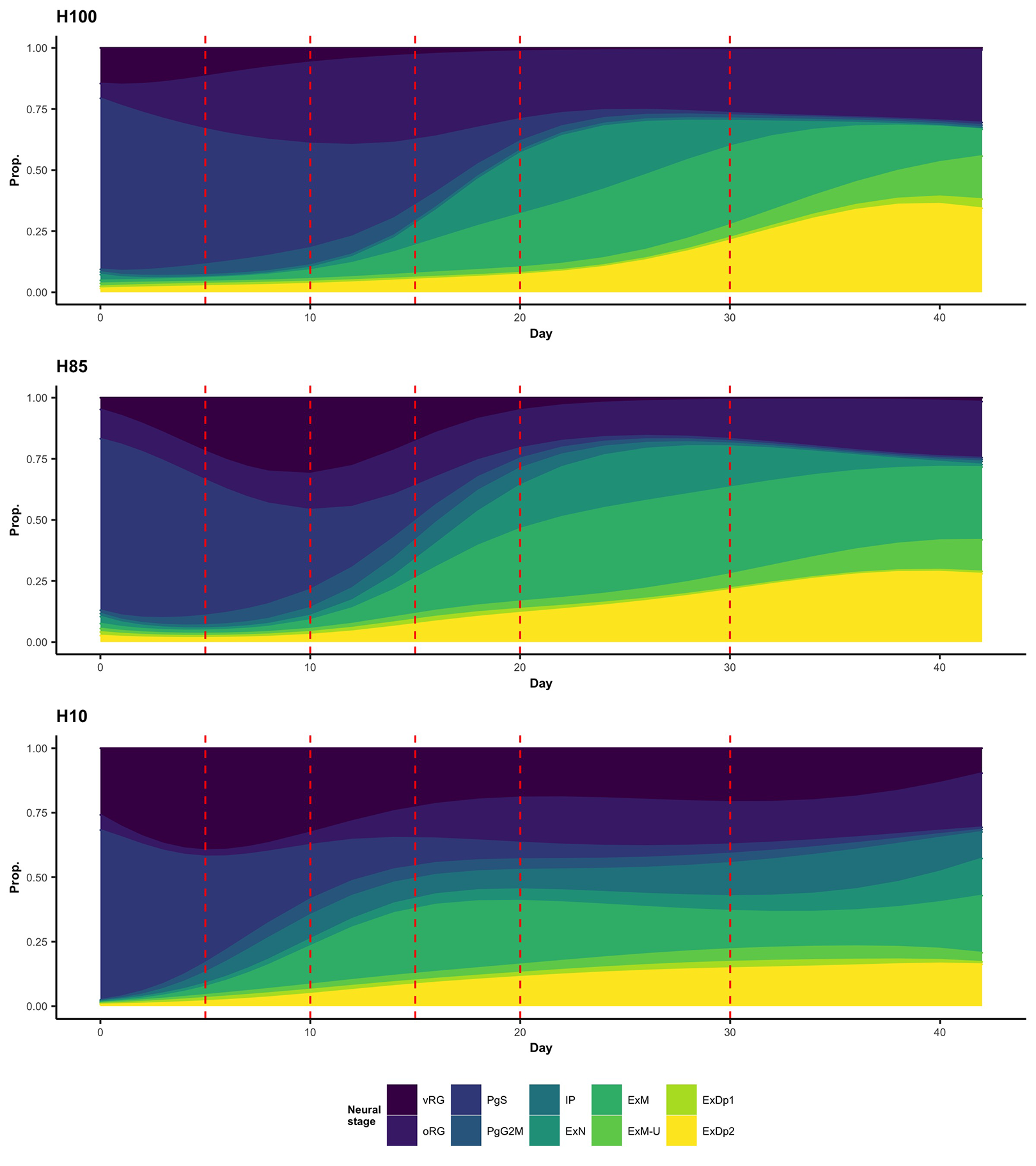

Supplement: S9 Fig — Expression data for H100, H85, and H10 respectively are deconvolved relative to the CoDEx reference dataset of annotated developing brain single cell expression. Deconvolution produces estimates of the relative proportions of reference cell-types present in the bulk data. Estimates are smoothed against time and plotted for each of H100 (top), H85 (middle), and H10 (bottom). (TIF) [file pcbi.1008778.s014.tif]

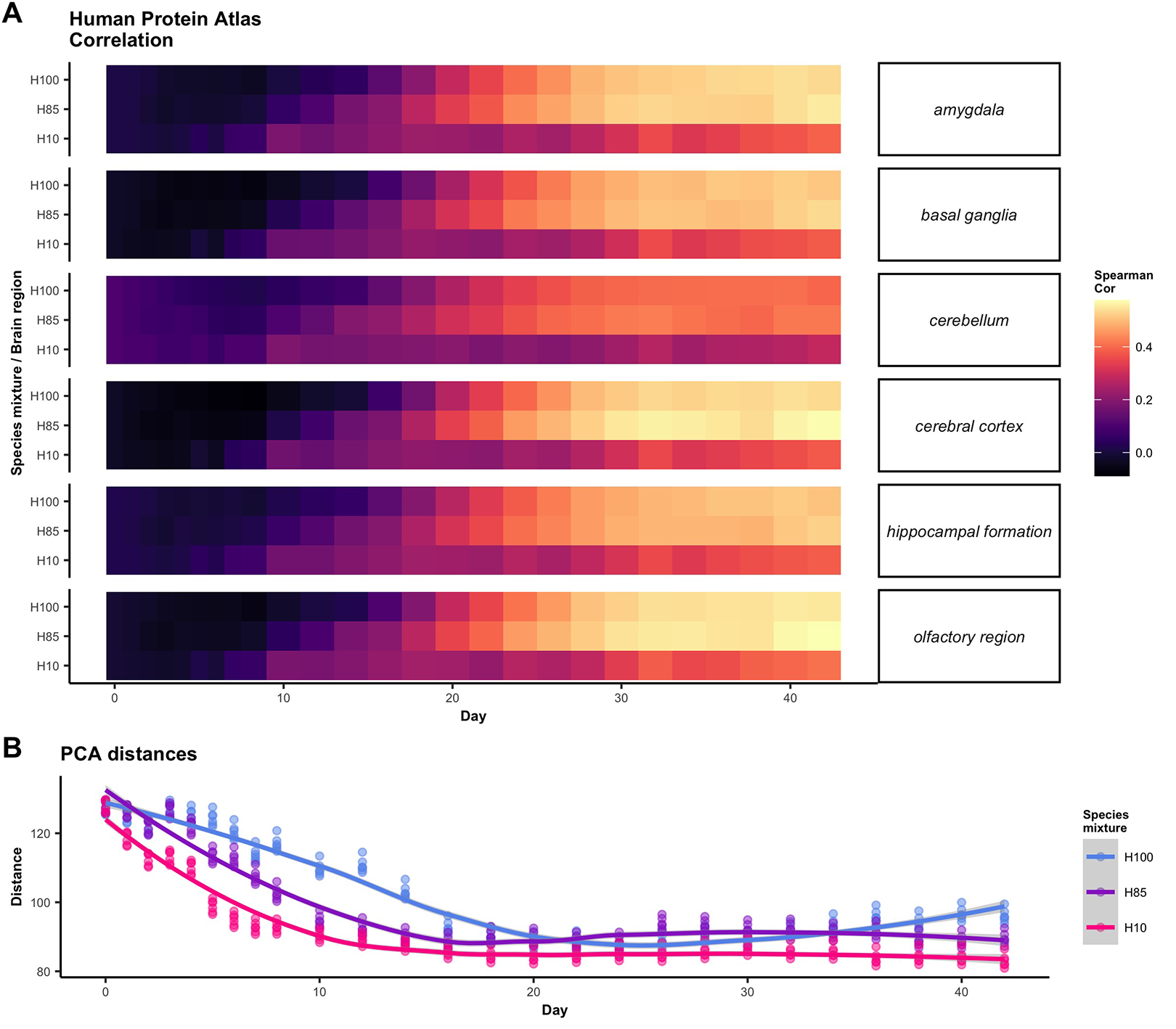

Supplement: S10 Fig — Correlations (Spearman) between fitted trends HPA data are calculated across the thirteen HPA regions. Calculations are performed on a subset of highly dynamic genes (see Materials and Methods). Dissimilarity (PCA-based distance, see Materials and Methods) between species mixtures and each of 6 HPA cell-types are computed for each day and smoothed to estimate a continuous dissimilarity metric over time. (TIF) [file pcbi.1008778.s015.tif]

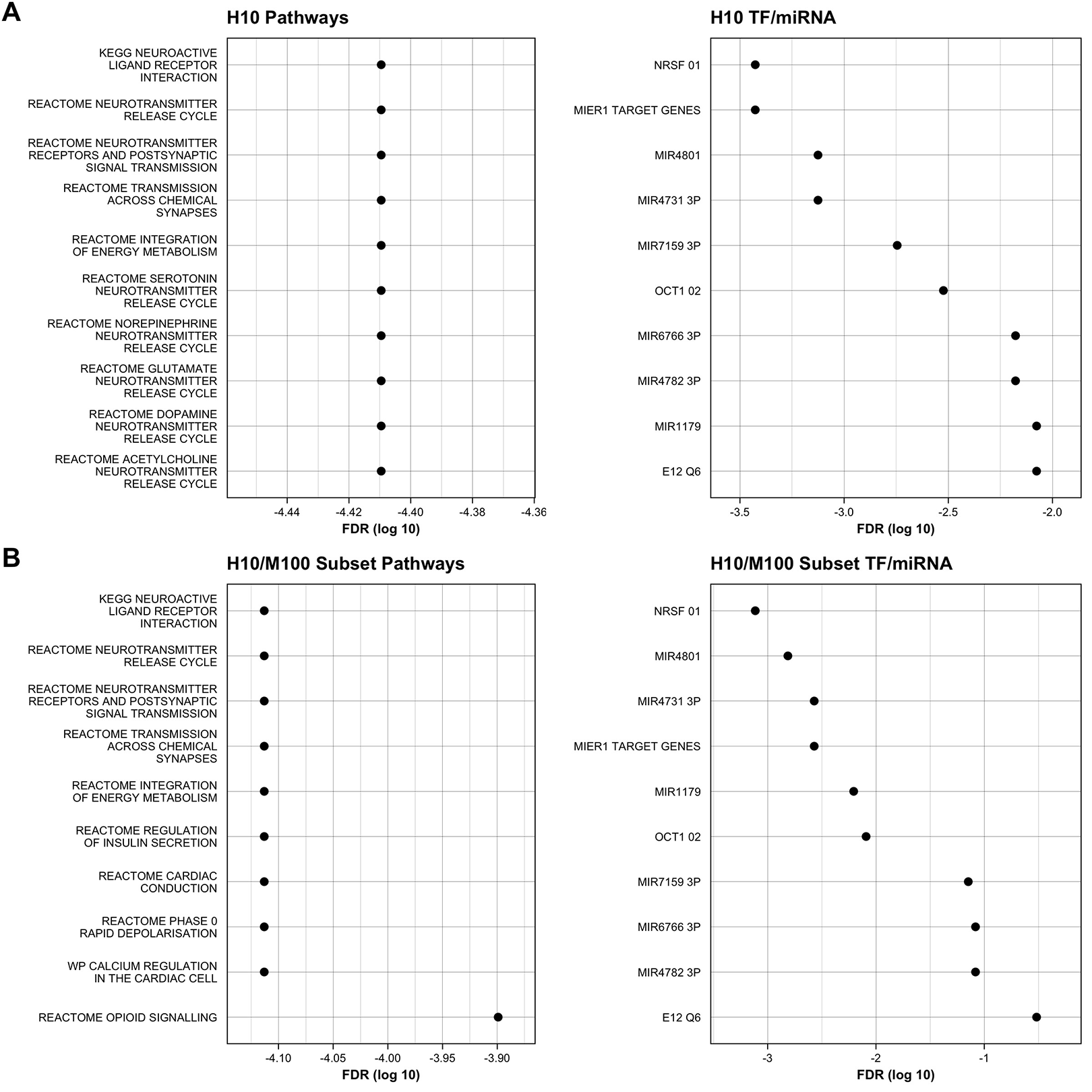

Supplement: S11 Fig — (A) Top pathways (left) and TFs/miRNAs (right) enriched for acceleration in H10 are plotted against their FDR corrected p-values. (B) Similar analysis is performed on M100 orthologs compared to H100 expression. Prior to plotting top pathways (left) and TFs/miRNAs (right), enriched terms are subset to include only those which are also significant (FDR corrected p-value ≤ 1e-2) in the above H10 comparison. (TIF) [file pcbi.1008778.s016.tif]
